# Reprogramming Transcriptional Networks via CREB1 Lactylation at K122 Activates HMGB1-Mediated NETosis and Chemoresistance

**DOI:** 10.7150/ijbs.131303

**Published:** 2026-05-18

**Authors:** Jia-Mei Wang, Fu-Ying Zhao, Qi Zhang, Ye Yuan, Bai-Qiang Li, Ning Liu, Jing-Jie Li, Chuan Liu, Hua-Qin Wang

**Affiliations:** 1 Department of Biochemistry and Molecular Biology, China Medical University, Shenyang, 110122, China.; 2 Department of Laboratory Medicine, The First Hospital of China Medical University, Shenyang, 110001, China.; 3 Department of Obstetrics and Gynecology, Shengjing Hospital of China Medical University, No. 36, Sanhao Street, Heping District, Shenyang, Liaoning Province 110004, China.; 4 Central Laboratory, Cancer Hospital of China Medical University, Liaoning Cancer Hospital & Institute, Cancer Hospital of Dalian University of Technology, Shenyang 110042, China.; 5 Department of University Hospital, Criminal Investigation Police University of China, Shenyang, 110854, China.

**Keywords:** CREB1 lactylation, cisplatin resistance, ovarian cancer, neutrophil extracellular traps (NETs), HMGB1, lipid nanoparticles

## Abstract

Cisplatin resistance remains a major obstacle in ovarian cancer treatment. While lactate-rich tumor microenvironments promote chemoresistance, the role of lysine lactylation (Kla) in this process remains poorly understood. Here, we identify CREB1 lactylation at K122 as a pivotal epigenetic driver of cisplatin resistance. Through quantitative lactyl-proteomics, we found CREB1 K122 as a hyperlactylated site specifically enriched in cisplatin-resistant ovarian cancer cells and patient tissues. This modification is dynamically regulated by the opposing activities of p300 (writer) and SIRT1 (eraser). Functionally, a lactylation-mimetic CREB1 mutant (K122Q) conferred robust resistance, enhancing cell survival and tumor growth, whereas a lactylation-deficient mutant (K122R) sensitized cells to cisplatin. Mechanistically, CUT&Tag analysis revealed that K122la remodels chromatin architecture, redistributing CREB1 binding from promoters to distal enhancers and substantially expanding its target repertoire. This transcriptional rewiring specifically activated neutrophil extracellular trap (NETosis) programs, with high mobility group box 1 (HMGB1) emerging as a key downstream effector. Lactylated CREB1 promotes HMGB1 transcription and subsequent exosomal secretion into the tumor microenvironment. Secreted HMGB1 then engages Toll-like receptor 4 (TLR4) on neutrophils to trigger NETosis, establishing a chemoprotective niche. Clinically, cisplatin-resistant patients exhibited elevated tumor K122 lactylation and serum exosomal HMGB1 levels. Most importantly, we developed a tumor-targeted lipid nanoparticle (LNP) system delivering a lactylation-deficient CREB1 K122R competitive peptide. This nanotherapeutic approach, particularly when combined with cisplatin, potently suppressed tumor growth *in vivo* and reduced serum exosomal HMGB1 levels, effectively reversing chemoresistance. Our work unveils the lactate-CREB1 K122la-HMGB1-NETs axis as a metabolic-epigenetic-immune driver of cisplatin resistance and provides a promising nanomedicine strategy for overcoming treatment resistance in ovarian cancer.

## Introduction

Ovarian cancer remains the most lethal gynecological malignancy, with >70% of patients diagnosed at advanced stages due to occult progression and a 5-year survival rate below 30%^1^, ^2^. Although platinum-based chemotherapy achieves initial therapeutic responses in 75-80% of cases^3^, ^4^, the majority (~70% of patients) ultimately develop refractory disease 5, 6, underscoring the critical need to decipher the underlying mechanisms of chemoresistance.

A prominent feature associated with treatment failure is the lactate-enriched acidic tumor microenvironment (TME), recognized as a contributor to tumor progression, immune evasion, and chemoresistance^7^-^9^. Beyond its role as a metabolic byproduct, lactate functions as a signaling molecule driving epigenetic reprogramming via lysine lactylation (Kla), a recently identified post-translational modification (PTM) regulating gene transcription^10^-^14^. Emerging evidence implicates Kla in medicating chemoresistance across malignancies, including modulation of autophagy in colorectal cancer^15^, promotion of DNA repair in gastric cancer via NBS1 lactylation 16, and enhancement of homologous recombination in breast cancer through MRE11 modification^9^. Despite these advances, the functional landscape of the lactylome in cisplatin resistance specific to ovarian carcinoma remains largely unexplored.

The transcription factor CREB1, frequently overexpressed in diverse cancers 17-21, orchestrates oncogenic programs via post-translational modifications. Despite its established role, the potential epigenetic regulation of CREB1 by lactylation and its consequent impact on chemoresistance is unknown. Our quantitative lactyl-proteomics (4D-FastDIA) identified CREB1 K122 as a hyper-lactylated site specifically within cisplatin-resistant ovarian cancer models, promoting the hypothesis that that lactate-induced CREB1 K122 lactylation (K122la) drives resistance by reprogramming transcriptional networks.

Intriguing preliminary observations further linked CREB1 to neutrophil extracellular traps (NETs), chromatin-based structures released during NETosis^22^, ^23^. Pathological NETosis is increasingly implicated in facilitating tumor progression and conferring chemoresistance^21^, ^24^-^28^. Both chemotherapeutic agents and tumor cells can directly induce NET formation, creating an immunosuppressive TME that protects cancer cells^29^-^31^. Whether a mechanistic axis connecting lactate metabolism, CREB1 lactylation, and NETosis underpins cisplatin resistance in ovarian cancer represents a significant gap in knowledge.

Herein, we elucidate that CREB1 K122la, dynamically modulated by the opposing enzymatic activities of p300 (writer) and SIRT1 (eraser), orchestrates epigenetic rewiring to transcriptionally activate High Mobility Group Box 1 (HMGB1). We further demonstrated that HMGB1 is subsequently secreted via exosomes into the TME, where it engages Toll-like receptor 4 (TLR4) on neutrophils to trigger NETosis, establishing a chemoprotective niche. Collectively, this work unveils the integrative lactate-CREB1 K122la-HMGB1-NETs axis as a novel metabolic-epigenetic-immune driver of cisplatin resistance in ovarian cancer, nominating actionable therapeutic targets within this pathway^32^.

Furthermore, as our understanding of tumor metabolic and epigenetic interplay deepens, targeting specific lactylation modifications has emerged as a promising strategy to overcome chemoresistance. Nanomedicine, particularly lipid nanoparticle (LNP) systems, offers considerable potential for precise intervention against tumor-specific pathways due to its superior drug delivery efficiency and tumor-targeting capabilities^33^, ^34^. However, the therapeutic potential of targeting lactylation via nanocarriers to reverse cisplatin resistance in ovarian cancer remains unexplored. Therefore, we developed a tumor-targeted LNP system designed to deliver a lactylation-deficient CREB1 K122R competitive peptide. This approach aims to specifically disrupt the transcriptional reprogramming driven by CREB1 K122la, thereby proposing a novel nanotherapeutic platform to clinically reverse cisplatin resistance.

## Methods

### Cell culture and cisplatin resistance induction

SKOV3 (RRID: CVCL_0C84) and A2780(RRID: CVCL_0134) human ovarian cancer cell lines were acquired from the American Type Culture Collection (ATCC, USA) via Procell Life Science & Technology Co., China. Cell lines were authenticated by short tandem repeat (STR) profiling and confirmed to be free of interspecies contamination. The cells were grown in RPMI1640 medium (Biological Industries, Israel) supplemented with 10% fetal bovine serum (Biological Industries, Israel) and 1% penicillin-streptomycin (Sigma-Aldrich, USA). They were maintained in a humidified incubator at 37 °C under 5% CO₂, and cells at the logarithmic growth phase were used for experiments. Cisplatin-resistant ovarian cancer cell lines (SKOV3/DDP and A2780/DDP) were established from parental SKOV3 and A2780 cells using a stepwise dose escalation protocol: The half-maximal inhibitory concentration (IC₅₀) of cisplatin in parental cells was determined via CCK-8 assay. Cells were chronically exposed to cisplatin starting at 10 nM, with the concentration incrementally increased by 50% every two weeks for 3-6 months until stable resistance was achieved. Resistant cells were maintained under continuous cisplatin pressure (final target concentration: 5-10 μM) with weekly cisplatin treatment (24-48 hours) to preserve the resistant phenotype.

### Clinical samples

Twelve patients aged 43 to 69 years who underwent surgical resection at the 1st Hospital of China Medical University were recruited in this study. The collected tissues were snap frozen in liquid nitrogen and stored in -80℃ freezer for further analysis. Based on whether recurrence occurred within six months after the first cisplatin-based chemotherapy regimen, the patients were assigned to two groups: a cisplatin-sensitive group (4 patients) and a cisplatin-resistant group (8 patients). None of the patients had received any chemotherapy or radiotherapy before surgery. In addition, a separate cohort of 50 ovarian cancer patients (25 cisplatin-sensitive and 25 cisplatin-resistant) was enrolled for blood sample collection. Serum samples were isolated and stored at -80°C for subsequent quantification of NETs and exosomal HMGB1. The project was approved by Institutional Review Board of the 1st Hospital of China Medical University (2024-199-2).

### Measurement of glucose consumption

Cells were seeded into 24-well plates at a density of 3 × 10^4^ cells per well in 0.5 mL of RPMI1640 medium. A control well containing only medium without cells was also prepared. After 24 h of culture, the glucose level in the culture medium was measured using a Glucose Assay Kit (Biovison, Milpitas, CA). The amount of glucose consumed was calculated by subtracting the measured glucose concentration from that of the control. The cells in each well were then counted, and the glucose consumption values were normalized to the cell number per well.

### Determination of the extracellular lactate

Cells cultured in 24-well plates were rinsed with PBS and treated with KRPH assay buffer (enriched with 0.2% BSA and 10 mM glucose) for 2 h. Next, supernatants were analyzed for their lactate content via an enzyme-coupled fluorescent assay using a L-Lactate Assay Kit (Cayman Chemical, Ann Arbor, MI) according to the manufacturer's instructions. For lactate quantification, an enzymatic cascade was utilized: lactate dehydrogenase mediates the oxidation of lactate to pyruvate coupled with the reduction of NAD^+^ to NADH. The resulting NADH reacts with a substrate to produce fluorescence, which was recorded at Ex/Em = 530/585 nm. To determine relative lactate release, the obtained data were adjusted based on respective cell numbers.

### Seahorse assay

The glycolytic function, represented by the extracellular acidification rate (ECAR), was evaluated in parental and cisplatin-resistant lines relying on the Seahorse XF24 platform (Seahorse Bioscience, North Billerica, MA, USA). Briefly, cells (1.5 × 10⁴ per well) were allowed to attach overnight in Agilent 24-well XF microplates. Prior to analysis, a gentle PBS wash was performed, and the medium was replaced with Seahorse incubation fluid containing 2 × 10^-3^ M L-glutamine and 1 × 10^-6^ M glucose. To prevent pH buffering and ensure accurate measurements, the plates were maintained at 37 °C without CO₂ for 60 min. Following the official Seahorse XF Glycolysis Rate Test protocol, basal ECAR was established before the sequential introduction of rotenone/antimycin A and 2-DG. Post-measurement, the Pierce Rapid Gold BCA Assay was employed to determine the live-cell protein concentration per well, which was utilized to normalize the ECAR data (presented as mean ± SD).

### CRISPR-Cas9 knockout of CREB1

CREB1-knockout (CREB1-KO) ovarian cancer cell lines were generated using the CRISPR-Cas9 system. Lentiviral particles containing CRISPR-Cas9 and single-guide RNAs (sgRNAs) targeting human CREB1 were commercially obtained from abm China. Two sgRNAs targeting exon 3 of the CREB1 gene were used:

sgRNA#1: 5'-CACCGTGGAGTTGGCACCGTTACAG-3'

sgRNA#2: 5'-AAACCTGTAACGGTGCCAACTCCAC-3'

Cells were infected with the lentivirus at an MOI of 20. After 48 h, infected cells were selected with 2 μg/mL puromycin for 7-10 days. Single-cell clones were isolated by limiting dilution, and CREB1 knockout was confirmed by western blot analysis using an anti-CREB1 antibody.

### 4D label-free proteomics analysis

The lactylation proteomics profiling of parental and cisplatin-resistant cell lines (SKOV3 and A2780) was performed in partnership with Jingjie PTM BioLabs (Hangzhou, China). Initially, cellular proteins were subjected to trypsin digestion and subsequent centrifugation to yield tryptic peptides. These peptides were then dissolved in NETN buffer. For the specific isolation of Klac-modified peptides, the samples were incubated with PTM-1404 antibody-conjugated beads (PTM Bio) under gentle rotation at 4 °C overnight. Following the enrichment process, the eluted peptides underwent fractionation via a 300Extend C18 column (Agilent, Santa Clara, USA) prior to comprehensive liquid chromatography-tandem mass spectrometry (LC-MS/MS) evaluation.

### Colony formation assay

For the plate colony formation assay, 200 cells/well were seeded into 12-well plates and cultured for 2 weeks. The cells were fixed with 4% paraformaldehyde for 15 min, then stained with 0.1% crystal violet. The colony number was determined under an optical microscopy.

### Western blot and immunoprecipitation

Cellular protein extraction was performed relying on a lysis buffer formulated with 1% Triton-X100, 2 mM EDTA, 150 mM NaCl, and 20 mM Tris-HCl, supplemented with a protease inhibitor cocktail (Sigma-Aldrich, Saint Louis, MO). Following concentration determination via a BCA assay kit, equal protein aliquots (20 μg) were resolved on 10% SDS-PAGE gels and subsequently electroblotted onto PVDF membranes (Millipore Corporation, Billerica, MA). For the immunoprecipitation (IP) assays, cell extracts were first pre-cleared utilizing protein A/G magnetic beads. The mixtures were then treated with specific antibodies and incubated at 4°C overnight. Post-incubation, the resulting immune complexes underwent three washes with the aforementioned lysis buffer prior to Western blotting analysis, which was performed with primary antibodies against D-Lactyl Lysine, HDAC1, HDAC3, SIRT1, SIRT3 (Jingjie); CREB1, p300/CBP, PCAF, Flag, and Myc antibodies (Cell Signaling Technology), and β-tubulin (Millipore).

### Measurement of plasma NETs and correlation analysis

Serum levels of neutrophil extracellular traps (NETs) were quantified by measuring MPO-DNA complexes. Briefly, 96-well plates were coated with anti-human myeloperoxidase (MPO) antibody (1 μg/mL; R&D Systems) overnight at 4°C. After blocking, plasma samples (diluted 1:2) were incubated for 2 h at room temperature. Subsequently, a peroxidase-labeled anti-human DNA antibody (Cell Death Detection ELISA kit, Roche) was used to detect DNA bound to MPO. The absorbance was measured at 405 nm. For correlation analysis, serum exosomal HMGB1 levels (determined by qRT-PCR) and plasma MPO-DNA values were subjected to Pearson correlation test.

### Nude mouse xenograft experiments

Female BALB/c-nu/nu mice (4-5 weeks of age) were acquired from Liaoning Changsheng Biotechnology Co., Ltd. The animals were grouped three per polyvinyl chloride (PVC) cage and maintained in an air laminar flow chamber. To ensure strict environmental control, the cages were equipped with sealed air filters and animal isolators. All experimental protocols were executed in compliance with the guidelines set by the Institutional Animal Care Committee of China Medical University. To establish the subcutaneous models, designated numbers of viable A2780/DDP and SKOV3/DDP cells were injected into the mice. Once the tumor volumes grew to approximately 100 mm³, LNP treatment was initiated. Throughout the 23-day therapeutic window, tumor dimensions were recorded at 3-day intervals. Following a total observation period of 28 days to monitor mouse health and tumor progression, the animals were euthanized. Finally, the subcutaneous xenografts were excised and photographically documented. At the endpoint, the mice were euthanized by cervical dislocation, subcutaneous tumors were carefully excised, photographed, and precisely weighed. We have specified the maximal permitted tumor burden (diameter not exceeding 1.5 cm) as stipulated by our ethics committee and confirmed that this limit was not exceeded in any animal during the study. All animal procedures were approved by and compiled with the guidelines of the Institutional Animal Care Committee of China Medical University (KT20240267).

### Cell viability assay

Cells were seeded at 1.0 × 10^4^ per well into 96-well plates. After adhesion, cells were treated with the indicated concentrations of cisplatin. Cell viability at 2 days was evaluated utilizing a CCK-8 Cell Proliferation Kit. Briefly, 10 μL of the CCK-8 solution was introduced into each well. Following a 3-hour incubation period at 37 °C, the optical density was quantified at 450 nm relying on a microplate reader.

### Tissue microarray and immunohistochemical staining

To evaluate pan-Klca expression, immunohistochemistry was performed on the tissue slices. The staining sequence started with antigen retrieval. To deactivate internal peroxidase activity, a 3% hydrogen peroxide treatment was applied, while 3% normal goat serum was used to block unspecific binding. Subsequently, the sections were incubated with the primary antibodies and then washed. Following incubation with a horseradish peroxidase-conjugated IgG secondary antibody, the final chromogenic detection was performed using fresh 3,3-diaminobenzidine.

### CUT&Tag

The CUT&Tag assay was performed on K122R and K122Q ovarian cancer cells. Cells were fixed (1% formaldehyde), permeabilized (0.05% digitonin), and bound to Concanavalin A beads. Primary antibodies (anti-CREB1 or IgG control) and secondary antibodies were sequentially incubated. Tagmentation was conducted using Tn5 transposase, followed by DNA purification and PCR amplification. Libraries were sequenced (Illumina NovaSeq), and data were analyzed via Bowtie2 alignment, MACS2 peak calling, and cluster Profiler enrichment. Quality metrics included fragment size selection, sequencing depth, and replicate consistency. The DNA libraries were sequenced on the Illumina PE150 platform to a depth of 20 G per sample. DNA Fragments are sequenced and analyzed by APExBIO Technology LLC. Visualization of called peaks was conducted using IGV v2.14.1.

### Neutrophil isolation

Peripheral blood neutrophils from healthy volunteers and cancer patients were harvested via gradient centrifugation utilizing a Solarbio separation kit (Cat# P9040, China). The collected neutrophils were temporarily maintained in 5% FBS-supplemented RPMI 1640 prior to immediate use.

### Adhesion assay

Human neutrophils (1 × 10⁶ cells/well) were seeded into 24-well plates and divided into three conditions: an untreated control group, a NET-generating group exposed to 20 nM PMA, and a NET-degrading group co-treated with 20 nM PMA and 100 U/mL DNase 1. Following a 4-h incubation, 1 × 10⁵ Dil-labeled cancer cells were added to interact with the intact neutrophils, intact NETs, or digested NETs. The co-culture lasted for 20 min, after which the wells were subjected to five PBS washes. Adhesion was evaluated by quantifying the retained cells across five randomly selected fields using a fluorescence microscope.

### Cell immunofluorescence staining

For immunostaining, PMA was employed to trigger NET release in 5 × 10⁴ neutrophils. Cellular fixation (4% paraformaldehyde), permeabilization (0.1% Triton X-100), and blocking (1% BSA) were sequentially performed. The NETs were first probed with an anti-MPO antibody (Proteintech, Cat# 22225-1-AP, China; 1:50) for 12-16 h at 4°C, followed by washing and a 1-h incubation at 37 °C with a CY3-labeled secondary antibody (Elabscience, Cat# E-AB-1010, China; 1:50). A subsequent round of washing and blocking preceded an overnight 4°C incubation with an anti-H3Cit primary antibody (Abcam, Cat# ab5103, USA; 1:1000). On the following day, slides were PBS-washed and treated with a FITC-labeled secondary antibody (Elabscience, Cat# E-AB-1014, China; 1:50) at 37 °C for 1 h. Nuclei were counterstained with DAPI (Solarbio, Cat# C0065, China). Finally, slides were preserved with a fluorescence quencher and captured via fluorescence microscopy.

### Preparation and characterization of LNP-CREB1(K122R)/DDP

**Peptide design**: The hyper-lactylation of CREB1 at K122 residue, catalyzed by the p300 histone acetyltransferase, is established as the critical mechanism driving chemoresistance in ovarian cancer. To therapeutically interfere with this activation step, we designed a lactylation-deficient CREB1 competitive peptide CREB1-K122R. The NLS-CREB1 K122R peptide (amino acid sequence: EILSRRPSYRRILNDLSSDAP) was designed to contain a nuclear localization signal (NLS, sequence: PKKKRKV) to direct the peptide to the nuclear compartment, where it competes for the lactylation site at K122 of CREB1. To enable efficient electrostatic encapsulation into cationic lipid nanoparticles, the lactylation-deficient competitive peptide (core sequence: EILSRRPSYRRILNDLSSDAP) was fused with an N-terminal SV40 nuclear localization signal (NLS: PKKKRKV). A tetra-glutamate tag (EEEE) was appended to the C-terminus via a flexible linker (GGGS) to confer a net negative charge to the peptide at physiological pH, thereby facilitating electrostatic interaction with the cationic lipid DOTAP during formulation. The full-length peptide sequence was: PKKKRKV-EILSRRPSYRRILNDLSSDAP-GGGS-EEEE. The peptide was synthesized at >95% purity, as confirmed by high-performance liquid chromatography (HPLC) and validated by mass spectrometry.

**Preparation of dual-loaded LNPs:** Cationic liposomal nanoparticles (LNPs) co-encapsulating the CREB1 K122R peptide and cisplatin (DDP), designated as LNP-CREB1(K122R)/DDP, were prepared using a thin-film hydration-sonication method with simultaneous dual-drug loading. Briefly, a lipid mixture composed of DOPE (20 mg), DOTAP (10 mg), cholesterol (10 mg), and DSPE-PEG-HA (1 mg) was dissolved in 10 mL of chloroform/ethanol (5:1, v/v). The organic solvent was evaporated under reduced pressure using a rotary evaporator at 40 °C to form a uniform thin lipid film.

The lipid film was then hydrated with 10 mL of nuclease-free ddH₂O containing both cisplatin (1 mg) and the NLS-CREB1(K122R) peptide (0.5 mg), adjusted to pH 6.0 to enhance electrostatic interaction between the cationic lipid DOTAP and the anionic peptide. Hydration was performed under gentle agitation at 60 °C for 1 hour to facilitate complete dispersion and drug incorporation into the aqueous core and lipid-water interface.

The resulting multilamellar vesicle suspension was sonicated (40 kHz, 300 W) for 10 min at 45 °C and subsequently extruded 10 times through a 0.22-μm polycarbonate membrane to yield homogeneous, unilamellar or oligolamellar nanoparticles. The final formulation was purified by size-exclusion chromatography using a Sephadex G-50 column equilibrated with PBS (pH 7.4) to remove unencapsulated cisplatin and free peptide.

Control nanoparticles-including empty LNPs, peptide-only loaded LNPs (LNP-CREB1(K122R)), and cisplatin-only loaded LNPs (LNP-DDP)-were prepared analogously by hydrating the lipid film with ddH₂O containing either peptide alone, cisplatin alone, or no cargo, respectively.

### Physicochemical characterization

**Size and zeta potential**: The hydrodynamic diameter, polydispersity index (PDI), and zeta potential of the LNPs were measured in triplicate using dynamic light scattering after appropriate dilution.

**Morphology**: The morphology of the LNPs was examined by transmission electron microscopy. A sample droplet was placed on a carbon-coated copper grid, negatively stained with 2% (w/v) phosphotungstic acid, and air-dried before imaging at 100 kV.

**Peptide encapsulation efficiency (EE) and drug loading (DL)**: The encapsulation efficiency of the peptide was determined using an ultrafiltration method. Briefly, the formulation was placed in an ultrafiltration centrifuge tube (e.g., with a 100 kDa molecular weight cutoff) and centrifuged at 4,000 ×g for 30 min. The concentration of the free peptide in the filtrate was quantified by HPLC (or UV-vis spectroscopy for higher accuracy) at the appropriate wavelength. The EE and DL were calculated using the following equations:

EE (%) = (W_total_peptide - W_free_peptide) / W_total_peptide × 100%

DL (%) = (W_encapsulated_peptide / W_total_lipids) × 100%

Results: EE_peptide = 94.2 ± 2.1%; DL_peptide = 1.45 ± 0.08%

### Serum stability assay

The *in vitro* stability of the NLS-CREB1 K122R peptide was assessed by incubation in 50% mouse serum at 37°C. The synthetic peptide (sequence: PKKKRKV-EILSRRPSYRRILNDLSSDAP-GGGS-EEEE) was dissolved in PBS and mixed with fresh mouse serum to a final concentration of 0.5 mg/mL in 50% serum. Peptide incubated in PBS alone served as a control. At predetermined time points (0, 4, 8, 12, 24, 36, and 48 h), aliquots were removed, mixed with acetonitrile to precipitate proteins, and centrifuged. The supernatants were analyzed by reversed-phase HPLC with UV detection at 214 nm. The percentage of remaining peptide was calculated relative to the 0 h time point. The half-life was calculated by fitting the degradation curve to a one-phase exponential decay model using GraphPad Prism. Experiments were performed in triplicate, and data are expressed as mean ± SD.

### Bioinformatics and statistical analysis

**RNA-seq and Differential Expression Analysis:** Raw sequencing reads (Illumina NovaSeq 6000, PE150) from K122R and K122Q ovarian cancer cells (n = 3 per group) were preprocessed using Trimmomatic (v0.39) to remove adapter sequences and low-quality bases. Clean reads were aligned to the human reference genome (hg38) using STAR (v2.7.9a) with default parameters. Gene-level read counts were quantified by featureCounts (v2.0.1) based on Ensembl annotation. Differential expression analysis was performed using the DESeq2 R package (v1.32.0). Genes meeting the thresholds of |log₂ fold change|>2 and adjusted *P*-value (Benjamini-Hochberg) < 0.05 were defined as differentially expressed genes (DEGs).

**CUT&Tag downstream and multi-omics analysis:** Following MACS2 peak calling in CUT&Tag data, genomic annotation of peaks and their distribution around transcription start sites (TSS) were analyzed and visualized using the ChIPseeker R package (v1.28.3). To define overlapping gene sets, the annotated peak-associated genes from CUT&Tag were intersected with the DEGs identified from the RNA-seq analysis.

**Functional enrichment analysis**: Gene Ontology (GO) and Kyoto Encyclopedia of Genes and Genomes (KEGG) pathway enrichment analyses were performed on specific gene sets (K122Q-specific, K122R-specific, or shared targets) using the clusterProfiler R package (v4.0.5). Statistical significance was evaluated via the hypergeometric test with Benjamini-Hochberg multiple testing correction. Terms with an adjusted *P*-value < 0.05 were considered significantly enriched.

**Gene set enrichment analysis (GSEA)**: GSEA was conducted using the clusterProfiler R package. All expressed genes were pre-ranked in descending order based on their log₂ fold change values derived from the DESeq2 analysis. Reference gene sets were obtained from the Molecular Signatures Database (MSigDB, v7.4). A normalized enrichment score (NES) was calculated for each pathway, and those with an adjusted *P*-value < 0.05 were considered statistically significant. All bioinformatics analyses were executed in the R statistical environment (v4.1.0).

**Motif enrichment analysis:** K122Q-specific enhancer peaks were defined as CUT&Tag peaks with a significant increase in binding in K122Q compared to K122R (fold change ≥ 2, *P*< 0.05) and located > 2 kb from the transcription start site. Motif enrichment analysis was performed using HOMER (v4.11) with default parameters. Random genomic regions matched for length and GC content were used as background. Enrichment of known transcription factor binding motifs was assessed using the hypergeometric test with Benjamini-Hochberg multiple testing correction.

**WGCNA co-expression network construction:** The identified WGCNA module genes were systematically intersected with the lactylated CREB1 binding targets obtained from our CUT&Tag sequencing data. Gene Ontology (GO) functional enrichment analysis of the gene module was performed using the clusterProfiler R package. For survival analysis, patients were stratified by the expression signature score of the hub network. Kaplan-Meier overall survival (OS) curves were generated, and statistical significance was calculated using the log-rank test. Transcriptomic and clinical survival data for ovarian cancer and renal cell carcinoma (RCC) subtypes (KIRC, KICH, and KIRP) were obtained from the TCGA database. Weighted Gene Co-expression Network Analysis was performed using the R package WGCNA. An appropriate soft-thresholding power was applied to build a scale-free network, followed by the construction of a topological overlap matrix (TOM) for gene clustering. Module identification, hub gene selection, and network topology visualization were conducted based on established methodological frameworks^35^.

### Statistical analysis

ANOVA and post hoc Dunnett's test were used to analyze the statistical significance differences in the most experiments, and the significance difference was defined as *P*<0.05. All experiments were repeated 3 times, and data were ex*p*ressed as mean±SD (standard deviation) of representative experiments.

## Results

### Metabolic reprogramming drives global lysine lactylation in cisplatin-resistant ovarian cancer

**Figure 1:**
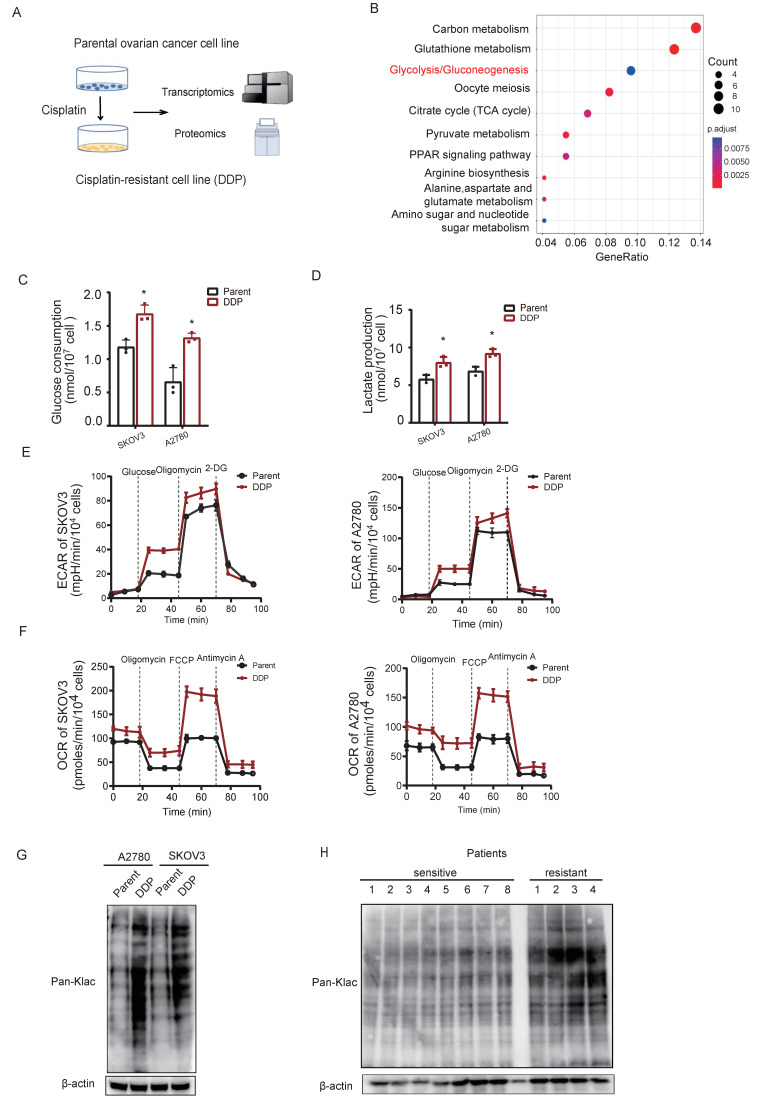

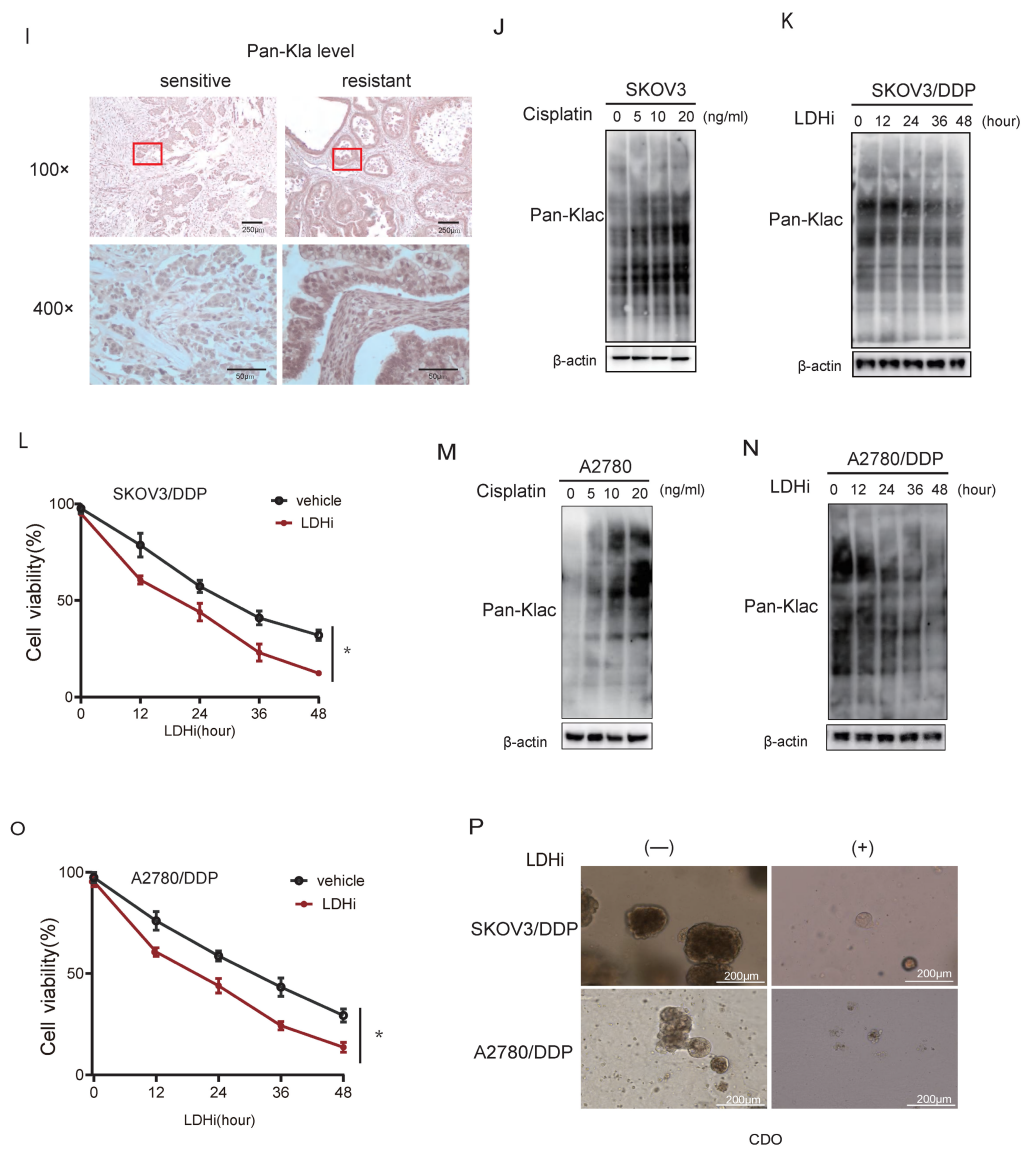
** Metabolic reprogramming drives global lysine lactylation in cisplatin-resistant ovarian cancer.** A, Schematic workflow of the multiomics analysis of cisplatin-resistant ovarian cancer cell lines. B, Kyoto Encyclopedia of Genes and Genomes (KEGG) analysis highlighted significantly upregulated proteins (log2FC >1) in cisplatin-resistant ovarian cancer cells compared with parental cells. C-D, Glucose consumption(C) and lactate production(D) in parental cells and cisplatin-resistant cells (DDP) of SKOV3 and A2780 ovarian cancer lines. Data presented as mean ± SD; * P < 0.05. E, Extracellular acidification rate (ECAR) tracing in SKOV3 and A2780 cells under metabolic stress (basal, glucose, oligomycin, 2-DG). F, Oxygen consumption rate (OCR) profiling in SKOV3 and A2780 cells under identical stress conditions. G, Western blot analysis of Pan-Klac expression in cisplatin-sensitive and cisplatin- resistant cell lines. H, Western blot analysis of Pan-Klac expression in cisplatin-sensitive and cisplatin-resistant patient samples. I, Representative case illustrating high versus low lactylation levels in cisplatin-sensitive and cisplatin-resistant human OV tissues assessed by immunohistochemistry (IHC) staining. J, Western blotting of total pan-Klac levels in SKOV3 cells treated with cisplatin for 1 week. K, Western blot analysis of pan-Klac levels in SKOV3/DDP cells treated with LDHi (10 × 10^-3^ M) at the indicated times. L, Determination of IC_50_ values in SKOV3/DDP cells cultured with LDHi for 48 h using the CCK8 assay. Data presented as mean ± SD; * P < 0.05. M, Western blotting of total pan-Klac levels in A2780 cells treated with cisplatin for 1 week. N,Western blot analysis of pan-Klac levels in A2780/DDP cells treated with LDHi (10 × 10^-3^ M) at the indicated times. O, Determination of IC50 values in A2780/DDP cells cultured with LDHi for 48 h using the CCK8 assay. Data presented as mean ± SD; * P < 0.05.P, Cisplatin-resistant patient-derived ovarian cancer organoids (CDOs) were treated with lactate dehydrogenase inhibitor (LDHi).

To model acquired cisplatin resistance, isogenic resistant derivatives (SKOV3/DDP, A2780/DDP) were established from parental ovarian cancer cell lines. Multi-omics profiling revealed concerted upregulation of glycolytic pathway components in resistant cells (Fig. 1A-B), indicative of metabolic reprogramming. Resistant cells exhibited enhanced Warburg effect phenotypes, including accelerated glucose consumption (Fig. 1C) and elevated lactate production (Fig. 1D). Seahorse analysis confirmed increased glycolytic flux (ECAR) and mitochondrial respiration (OCR) (Fig. 1E-F), consolidating hyper-glycolysis as a metabolic hallmark of cisplatin resistance.

Given lactate's established role in promoting Kla^36^, we assessed global Kla levels. Immunoblotting demonstrated significantly elevated pan-Kla in resistant versus parental cells (Fig. 1G). This phenotype was recapitulated *in vivo*, with cisplatin-resistant patient tissues exhibiting increased pan-Kla by immunoblotting (Fig. 1H) and immunohistochemistry (Fig. 1I). Dose-dependent lactylation induction was further observed in SKOV3 cells exposed to escalating cisplatin concentrations under lactic acid supplementation (Fig. 1J).

To establish functional causality, lactate production was inhibited using a lactate dehydrogenase inhibitor (LDHi). Treatment with LDHi substantially suppressed global lysine lactylation (Kla) levels in SKOV3/DDP cells (Fig. 1K) and concomitantly reduced cell viability (Fig. 1L). Consistently, treatment of A2780 cells with cisplatin increased global Kla levels (Fig. 1M). Furthermore, LDHi treatment of A2780/DDP cells reduced Kla levels (Fig. 1N) and consequently enhanced their sensitivity to cisplatin (Fig. 1O). In cisplatin-resistant patient-derived organoids (CDOs), LDHi abrogated growth within 72 hours (Fig. 1P), directly linking lactate-driven lactylation to chemoresistance maintenance.

### CREB1 K122la as a cisplatin-resistance specific epigenetic driver

**Figure 2:**
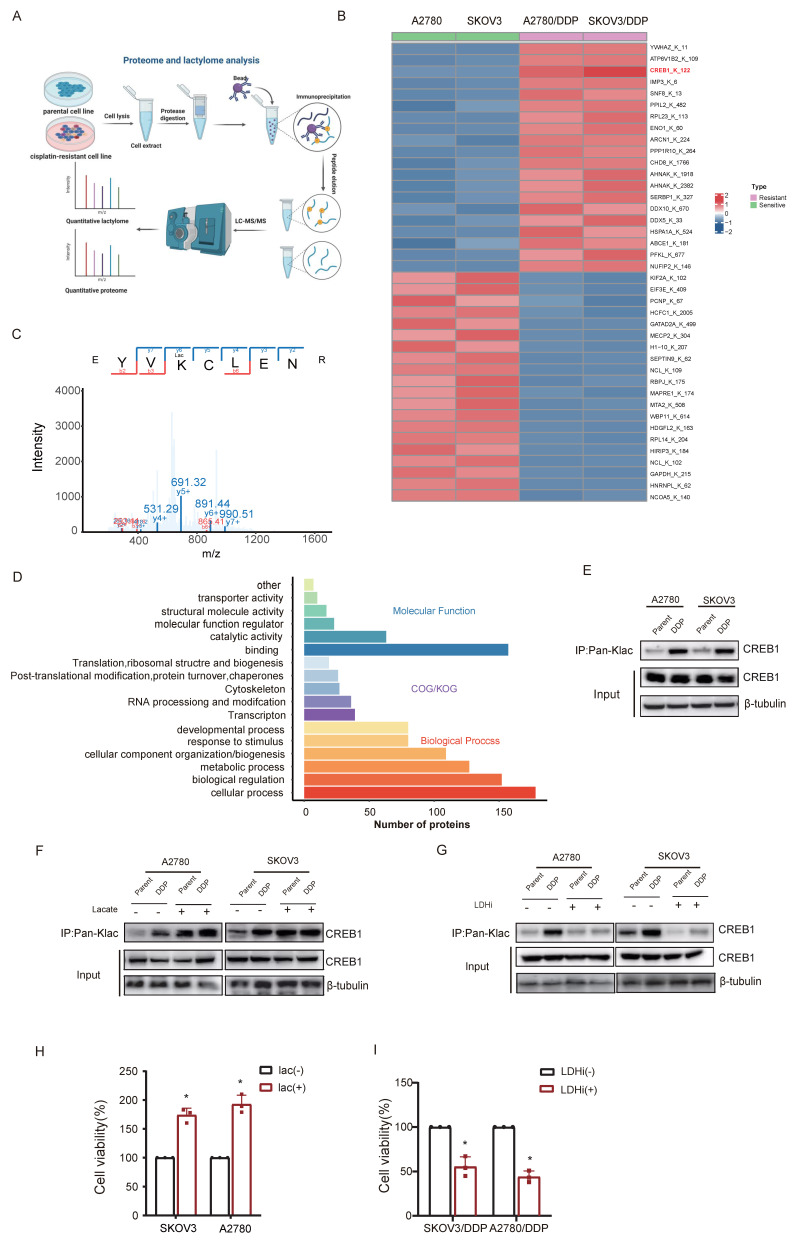
** CREB1 K122la as a cisplatin-resistance specific epigenetic driver.** A, Quantitative lactyl-proteomics (4D-FastDIA) profiling of cisplatin-sensitive vs. resistant ovarian cancer cells. B, Heatmap of the top 20 hyper-/hypo-lactylated proteins and sites ranked by fold-change. C, Mapping of CREB1 lactylation sites in cisplatin-resistant cells cells identified by liquid chromatography‒mass spectrometry (LC‒MS). D, Functional profiling of genes undergoing Lysine Lactylation. E, Co-immunoprecipitation (Co-IP) with pan-Kla antibody confirms CREB1 lactylation exclusively in cisplatin-resistant cells. F, Co-IP with pan-Kla antibody confirms lactate challenge boosts CREB1 K122la levels in resistant cells. G, Co-IP with pan-Kla antibody confirms LDHi reduced CREB1 K122la levels. H, CCK-8 assay demonstrating that lactate pretreatment enhances cisplatin resistance in sensitive cells. I, CCK-8 assay demonstrating that LDHi treatment restores cisplatin sensitivity in resistant cells.

Quantitative lactyl-proteomics (4D-FastDIA) comparing sensitive and resistant ovarian cancer cell identified 598 differentially lactylated proteins harboring 811 modified sites (Fig. 2A), with the top 20 hyper-/hypo-lactylated candidates profiled (Fig. 2B). Functional enrichment revealed nuclear predominance of lactylated proteins (Fig. 2C), suggesting transcriptional regulatory roles. In the lactyl-proteomics dataset, CREB1 was identified as one of the transcription factors exhibiting the most pronounced differential lactylation levels, with its K122 residue ranking among the top 3 sites in modification abundance in chemoresistant samples. Moreover, given the well-established central role of CREB1 in transcriptional regulation in cancer, it was prioritized as the primary candidate for further investigation. Notably, CREB1 emerged as the most dysregulated transcription factor, with site-specific mapping unambiguously identifying K122 as its dominant lactylation site in resistant cells (Fig. 2D).

Co-immunoprecipitation with pan-Kla antibodies confirmed CREB1 lactylation exclusively in resistant models (Fig.2E). Lactate challenge markedly amplified CREB1 K122la levels (Fig. 2F), while pharmacological lactate depletion suppressed this modification (Fig. 2G). We next investigated whether acute modulation of CREB1 lactylation directly impacts cisplatin sensitivity. In cisplatin-sensitive cells, pretreatment with 20 mM sodium lactate for 24 h significantly enhanced cell viability upon subsequent exposure to 20ng/mL cisplatin for 48 h, as determined by CCK-8 assay (Fig. 2H). Conversely, in cisplatin-resistant cells, treatment with 10 mM lactate dehydrogenase inhibitor (LDHi) for 48 h markedly re-sensitized the cells to 5 μg/mL cisplatin, leading to reduced viability compared to untreated resistant controls (Fig. 2I). These data establish CREB1 K122 lactylation as a lactate-dependent, resistance-specific epigenetic signature.

To further determine whether glycolysis-derived lactate directly fuels CREB1 lactylation, we treated cisplatin-resistant SKOV3/DDP and A2780/DDP cells with the glycolytic inhibitor 2-deoxyglucose (2-DG). As shown in Supplementary Fig. 1 (A-B), 2-DG treatment significantly reduced both glucose consumption and lactate production. Concomitantly, CREB1 lactylation levels were markedly decreased upon 2-DG treatment (Supplementary Fig. 1 C), indicating that glycolytic flux is required to sustain this modification. We next examined whether lactate serves as a direct metabolic substrate or a non-specific signaling molecule for CREB1 lactylation. Competition experiments using other short-chain fatty acids (acetate, butyrate) revealed that co-treatment with excess acetate or butyrate did not attenuate lactate-induced CREB1 lactylation, whereas inhibition of lactate dehydrogenase (LDHi) completely abrogated it (Supplementary Fig. 1D). These results indicate that the lactyl group donor is specifically derived from lactate metabolism rather than through non-specific acyltransferase activities.

We next compared the effects of equimolar concentrations of pyruvate and lactate on CREB1 lactylation. While lactate rapidly induced CREB1 lactylation, pyruvate did not (Supplementary Fig. 1E), suggesting that the conversion of pyruvate to lactate via LDH is a critical step. Collectively, these results establish that glycolytically derived lactate, rather than other intermediate metabolites, serves as the key precursor for CREB1 lactylation in cisplatin-resistant ovarian cancer cells.

### Bidirectional control of CREB1 K122la dynamics by p300 and SIRT1

**Figure 3:**
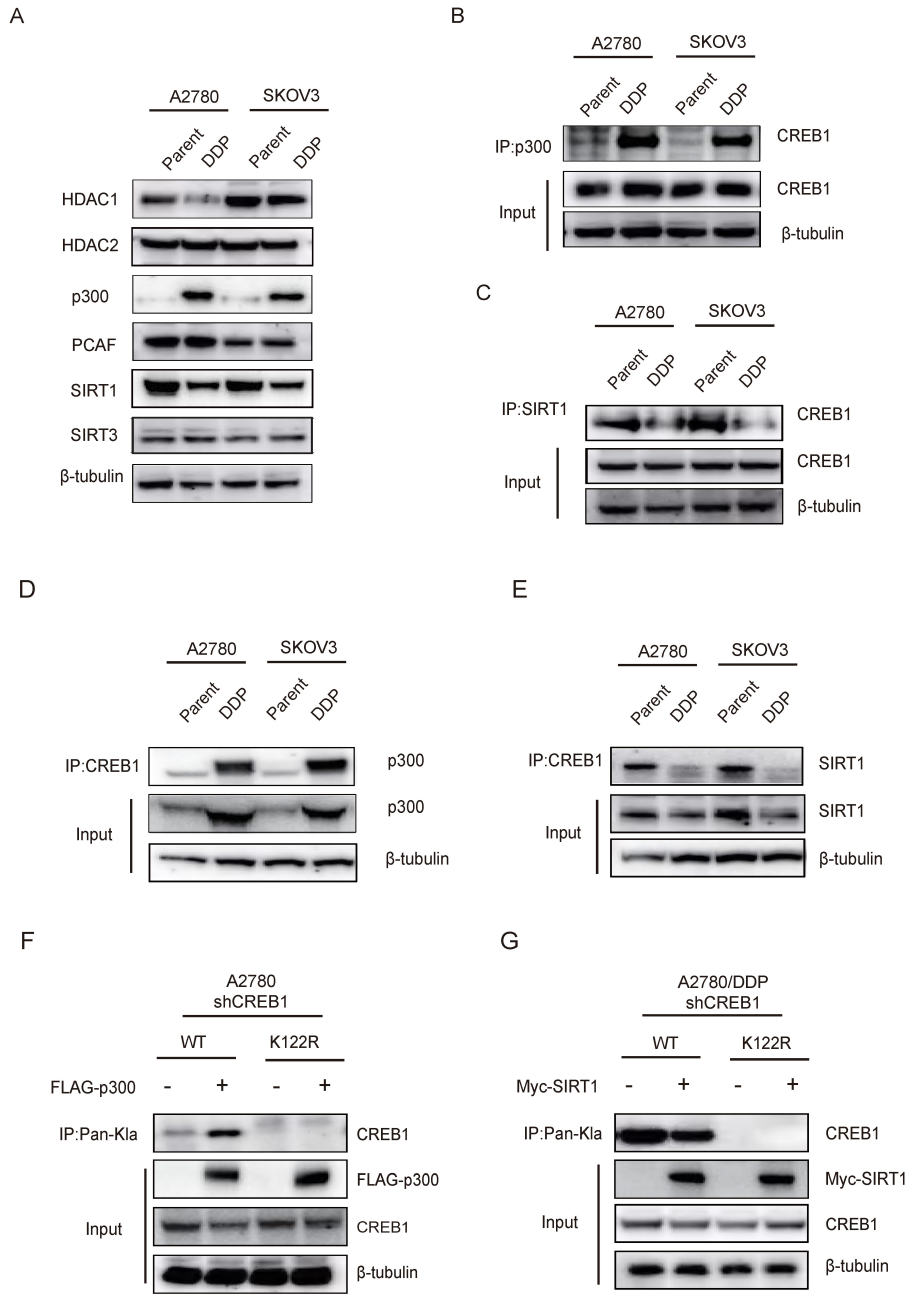
** Bidirectional control of CREB1 K122la dynamics by p300 and SIRT1.** A, Western blot analysis of Lactylation-modifying enzymes in cisplatin-sensitive and cisplatin-resistant ovarian cancer cells. B, Co-Immunoprecipitation (Co-IP) analysis of CREB1-p300 binding in cisplatin-sensitive and cisplatin-resistant ovarian cancer cells. C, Co-IP analysis of CREB1-SIRT1 binding in cisplatin-sensitive and cisplatin-resistant ovarian cancer cells. D-E, Co-IP analysis of p300-CREB1 binding (D)and SIRT1-CREB1 binding (E) in cisplatin-sensitive and cisplatin-resistant ovarian cancer cells. F-G, Co-IP analysis of binding interactions between lactylation-deficient mutant CREB1(K122R) and p300/SIRT1.

Interrogation of lactylation-modifying enzymes revealed p300 upregulation concurrent with HDAC1 and SIRT1 suppression in resistant cells (Fig. 3A). Co-immunoprecipitation assays confirmed interaction of CREB1 with p300 (Fig. 3B, D), as well as with SIRT1 (Fig. 3C, E), implicating p300 as a direct writer enzyme enhancing CREB1 K122la, and SIRT1 as an eraser suppressing this mark.

Reconstitution experiments in CREB1-knockout cells demonstrated that exogenous p300/SIRT1 modulated K122la exclusively in WT CREB1 (Fig. 3F-G). Conversely, K122R mutation abrogated physical interactions between CREB1 and both enzymes (Fig. 3F-G), confirming K122 as the specific regulatory site. These results establish p300 and SIRT1 as druggable direct bidirectional regulators of CREB1 K122la.

### CREB1 K122la confers cisplatin resistance *in vitro* and *in vivo*

**Figure 4:**
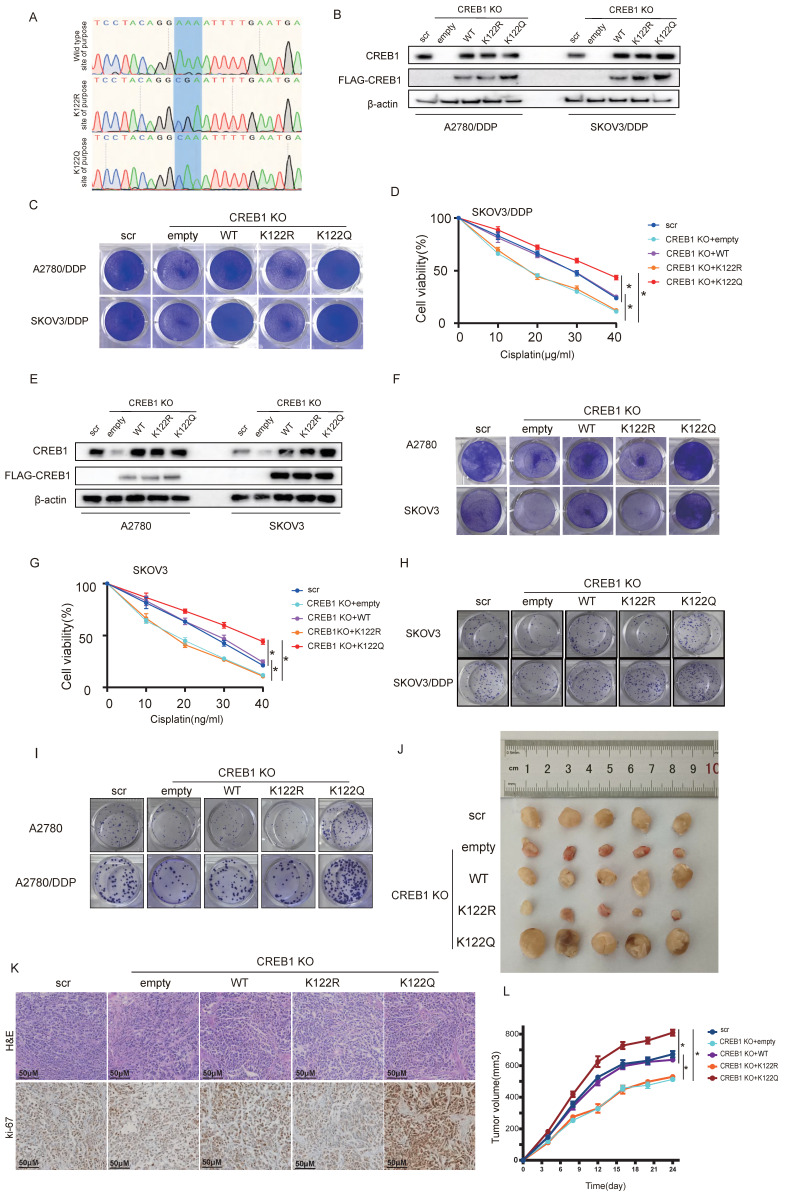
** CREB1 K122la confers cisplatin resistance *in vitro* and *in vivo*.** A, Structural models of CREB1 mutants. B, Validation of CREB1 knockout and K122Q/K122R mutant reconstitution in cisplatin-resistant cells (SKOV3/DDP, A2780/DDP). C, Crystal violet staining of cell survival in CREB1 Wild-Type and K122R/K122Q mutant cells under cisplatin treatment in cisplatin-resistant cells. D, Analysis of cell survival capacity in CREB1 Wild-Type and K122R/ K122Q mutant cells under gradient cisplatin treatment in SKOV3/DDP cells. Data presented as mean ± SD; * P < 0.05. E, Validation of CREB1 knockout and K122Q/K122R mutant reconstitution in cisplatin-sensitive cells (SKOV3, A2780). F, Crystal violet staining of cell survival in CREB1 Wild-Type and K122R/K122Q mutant cells under cisplatin treatment in cisplatin-sensitive cells. G, Analysis of cell survival capacity in CREB1 Wild-Type and K122R/ K122Q mutant cells under gradient cisplatin treatment in SKOV3 cells. Data presented as mean ± SD; * P < 0.05. H-I, Clone formation assay for anchorage-independent growth capacity of CREB1 Wild-Type and K122R/Q mutant cells. J, A subcutaneous tumor model in BALB/c-Nude mice was utilized to investigate the tumorigenic ability of cell lines subjected to CREB1 K122R/K122Q mutant. K, H&E staining and Ki-67 immunohistochemistry for quantitative assessment of subcutaneous tumor progression in BALB/c-Nude mice. L, A subcutaneous tumor model in BALB/c-Nude mice was utilized to investigate the tumorigenic ability of cell lines subjected to different treatments, while monitoring tumor volume changes over time. Data presented as mean ± SD; * P < 0.05.

To study the functional impact of CREB1 K122 lactylation, CRISPR-mediated CREB1 knockout resistant cells were reconstituted with wild-type (WT), lactylation-deficient (K122R), or lactylation-mimetic (K122Q) mutants (Fig. 4A-B). K122Q-CREB1 conferred profound resistance to cisplatin, significantly enhancing viability. Conversely, K122R mutation increased cellular sensitivity to cisplatin, resulting in significantly elevated mortality under cisplatin treatment (Fig. 4C-D).

This phenotype generalized to K122Q/K122R mutants in cisplatin-sensitive cells (Fig. 4E-G). Critically, K122Q enhanced clonogenic survival while K122R abated colony formation (Fig. 4H-I), establishing K122la as a master regulator of tumor cell fitness under cisplatin pressure.

*In vivo*, K122Q-expressing xenografts displayed accelerated tumor growth and cisplatin evasion, whereas K122R tumors were suppressed (Fig. 4J). Histopathological analysis demonstrated invasive fronts and elevated ki-67 positivity in K122Q tumors (Fig. 4K), establishing CREB1 K122la as a bona fide driver of cisplatin resistance.

### Transcriptional rewiring by CREB1 K122la activates NETosis programs

**Figure 5:**
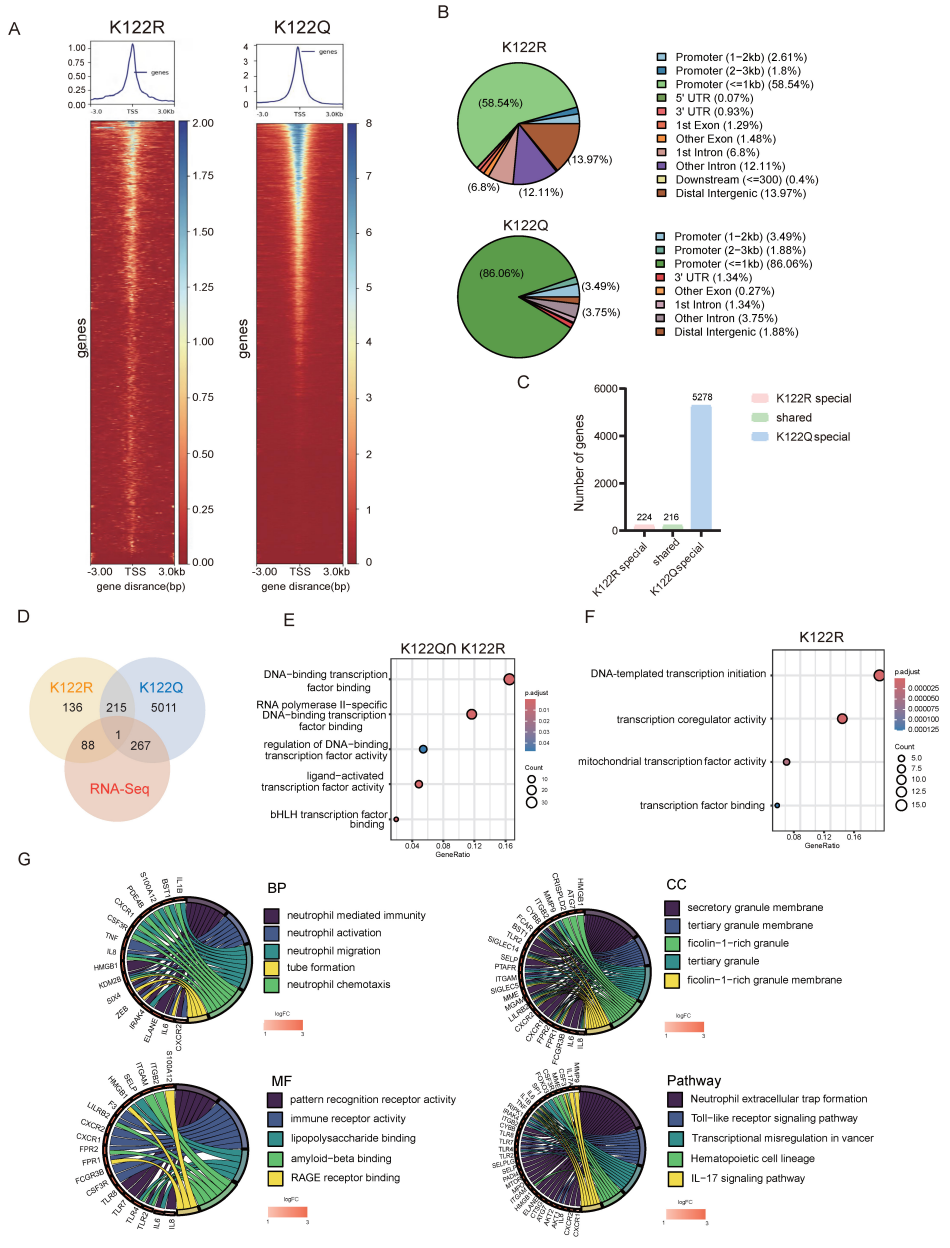

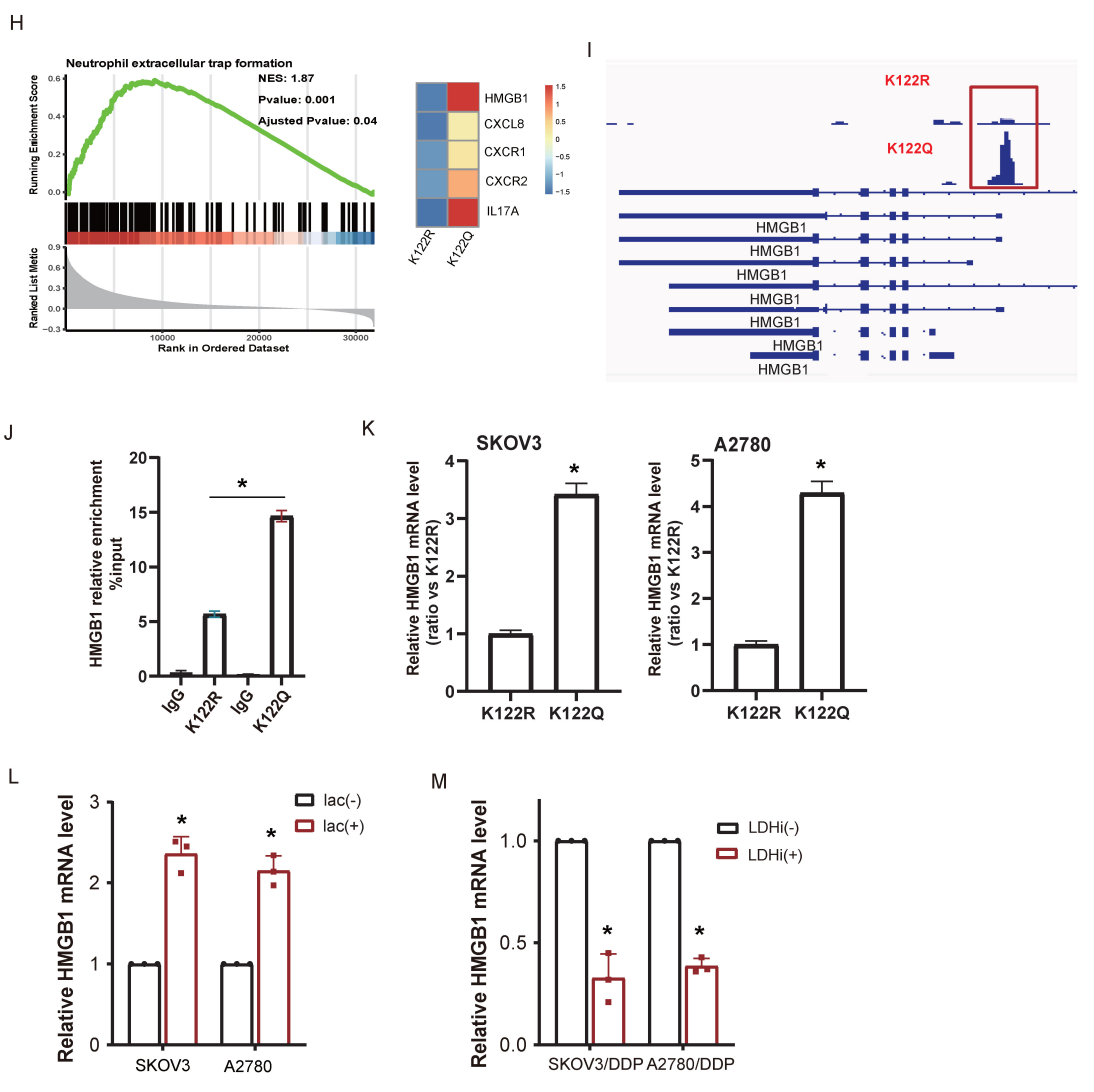
**Transcriptional rewiring by CREB1 K122la activates NETosis programs.** A, CUT&TAG was used to measure chromatin opening in K122R and K122Q cells. B, The distribution of CREB1 on the genome in K122R and K122Q cells. C, Bar plot showing genes with promoters marked by increases in only CREB1 K122R cells, increases in both CREB1 K122R and K122Q cells, or increases in only CREB1 K122Q cells. D, Combination of CUT&Tag and RNA-seq database to identify the potential downstream targets of CREB1 K122R/K122Q. E, KEGG pathway analysis of genes bound to CREB1 K122R and K122Q.F, KEGG pathway analysis of genes bound to CREB1 K122R. G, GO enrichment analysis of biological functions for genes specifically bound by CREB1 K122Q Mutant. H, GSEA enrichment analysis of biological functions and pathways for transcriptional regulatory targets specifically bound by CREB1 K122Q Mutant. I, IGV map of CUT&TAG to demonstrate the transcriptional open state of HMGB1 in K122R and K122Q cells. J, ChIP-qPCR assays of HMGB1 occupancy rates in the promoter region of CREB1 K122R/K122Q. Data presented as mean ± SD; * P < 0.05.K, RT-qPCR was used to detect the HMGB1 mRNA level in CREB1 K122R/K122Q cells. Data presented as mean ± SD; * P < 0.05. L, RT-qPCR analysis of *HMGB1* mRNA in SKOV3 and A2780 cells treated with lactate (20 mM) for 24 h. M, RT-qPCR analysis of *HMGB1* mRNA in SKOV3/DDP and A2780/DDP cells treated with LDHi (10 mM) for 48 h. Data are presented as mean ± SD; * P < 0.05 versus vehicle control.

CUT&Tag profiling revealed that K122Q dramatically enhanced chromatin accessibility (Fig. 5A) and redistributed CREB1 binding-genome wide: K122Q invaded distal enhancers (13.97% >2 kb upstream) and intronic regions (12.11%), contrasting with K122R's promoter-restricted occupancy (86.06% within 1 kb of TSS), (Fig. 5B). Critically, K122Q substantially expanded the CREB1 regulon, binding 5,278 target genes-significantly more than the 224 genes bound by K122R (Fig. 5C).

Integrated transcriptomics identified 267 K122Q-specific differentially expressed genes (DEGs) compared to 88 DEGs associated with K122R (Fig. 5D). Functional dissection revealed that shared targets were enriched in core transcriptional machinery functions (Fig. 5E), while K122Q-exclusive targets dominated neutrophil activation/immunity pathways (Fig. 5G). Notably, pathway analysis convergently implicated NETs formation and TLR signaling (Fig. 5G), with GSEA confirming robust enrichment of NETosis-related pathways (Fig. 5H).

Among K122Q-bound NETs effectors, HMGB1 emerged as a top candidate. CREB1 K122la directly facilitated physical HMGB1 interaction (Fig. 5I), with ChIP-qPCR confirming enhanced recruitment by CREB1 K122Q mutant (Fig. 5J). qRT-PCR analysis demonstrated elevated HMGB1 expression in K122Q mutant cells (Fig. 5K). These results position HMGB1 as a key mediator linking lactylation to immune evasion.

To further explore whether this regulatory axis is dynamically responsive to upstream metabolic cues, we examined HMGB1 expression following acute modulation of lactate levels. Treatment of cisplatin-sensitive SKOV3 and A2780 cells with lactate (20 mM, 24 h) significantly upregulated HMGB1 mRNA, while treatment of cisplatin-resistant SKOV3/DDP and A2780/DDP cells with the lactate dehydrogenase inhibitor (LDHi, 10 mM, 48 h) markedly downregulated its expression (Fig. 5L-M). These data demonstrate that the CREB1 K122la-HMGB1 axis is not only a stable feature of established resistance but also rapidly responds to changes in lactate availability, further strengthening the causal link between lactate metabolism, epigenetic modification, and downstream effector activation.

To assess whether K122Q binding reflects non-specific DNA binding, we performed motif enrichment analysis on K122Q-bound enhancers using HOMER. Compared to size-matched random genomic regions, K122Q-bound enhancers showed highly significant enrichment of the canonical CRE motif (Fold Enrichment=13.7, *P*=1.0 × 10^-125^), with 42.5% of enhancers containing the motif versus 3.1% of background regions (Supplementary Fig. 2). These results indicate that even in the lactylation-mimetic state, CREB1 binding remains sequence-specific.

### Orchestration of HMGB1-dependent NETosis by CREB1 K122la

**Figure 6:**
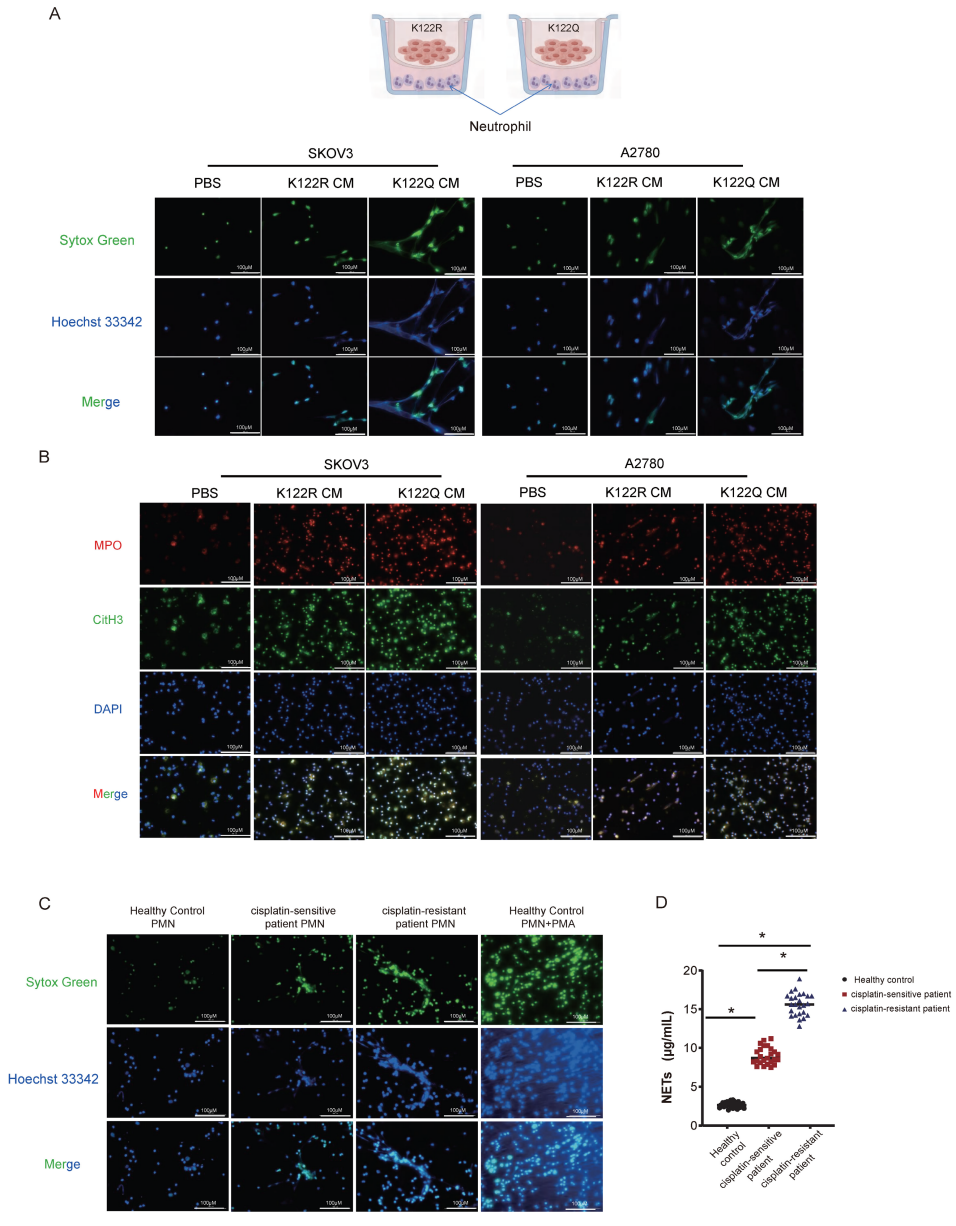

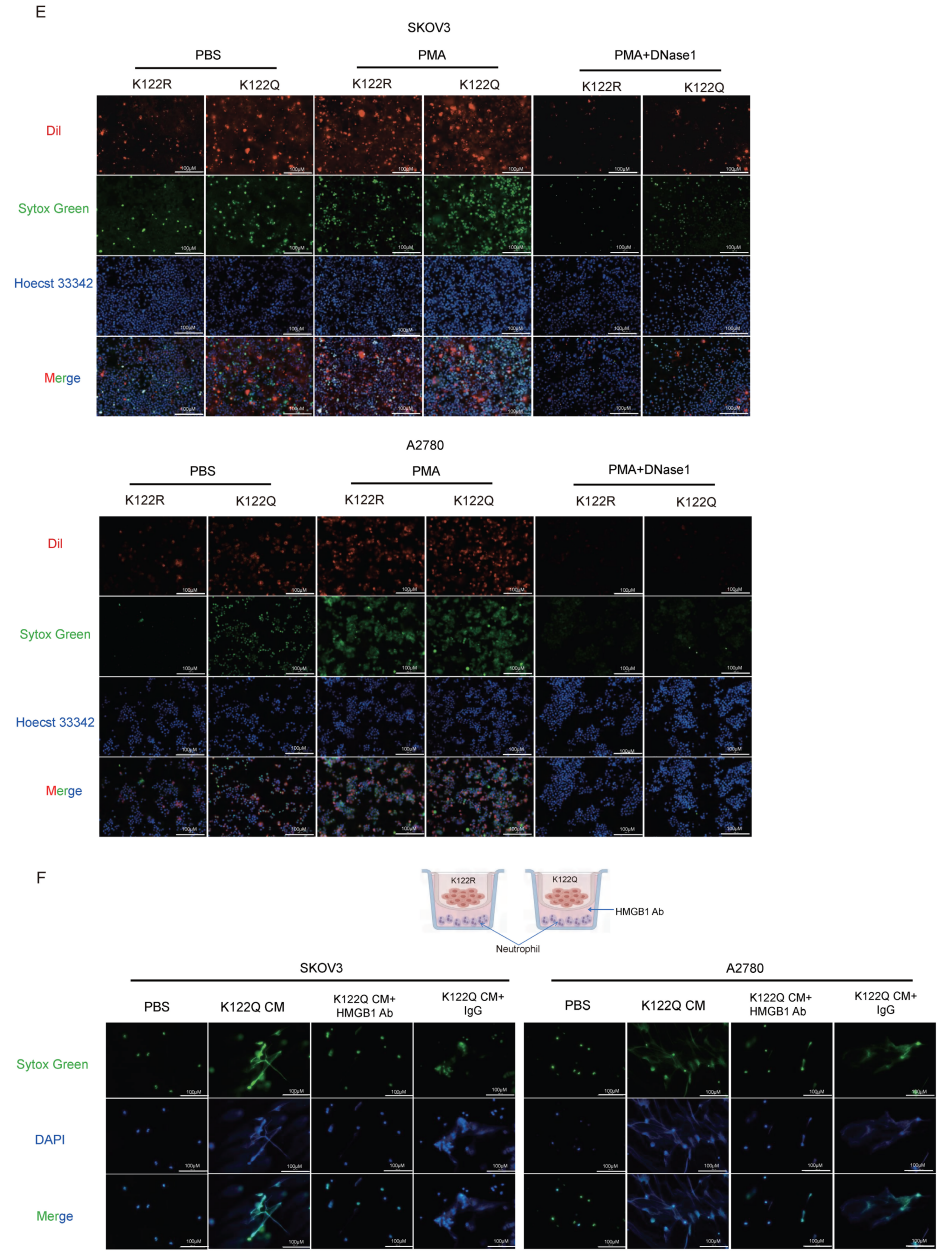
**Orchestration of HMGB1-dependent NETosis by CREB1 K122la.** A, Schematic diagram of the Transwell co-culture system for ovarian cancer cells (upper chamber) and neutrophils (lower chamber). Detection of Neutrophil Extracellular Trap (NET) Formation in body weightPMNs after Co-culture.B, IF for MPO and H3Cit in neutrophils and NETs following coculture with CM from CREB1 K122R/K122Q cells. C, Assessment of NETs Formation in peripheral blood neutrophils from cisplatin-sensitive and cisplatin-resistant ovarian cancer patients. D, ELISA quantification of NETs Levels in peripheral blood from cisplatin-sensitive and cisplatin-resistant ovarian cancer patients (n = 25 per group). E, Adhesion of K122R/K122Q cells within NETs, as opposed to intact neutrophils or NETs digested with DNase 1. F, Analysis of NETs formation in co-culture systems following HMGB1 neutralizing antibody intervention.

Given transcriptional links to NETosis pathways, we tested whether CREB1 K122la functionally regulates NETs formation. Compared with conditioned medium from K122R-mutant cells or controls, conditioned medium from K122Q-mutant cells potently induced NETosis in co-cultured neutrophils (Fig. 6A), validated by elevated MPO/H3Cit (Fig. 6B). Clinically, neutrophils from cisplatin-resistant patients exhibited maximal NETosis activity (Fig. 6C), with elevated serum NETs biomarkers s in resistant cohorts (Fig. 6D, n = 25 per group), confirming clinical relevance.

Mechanistically, K122Q-mutant cells showed enhanced adhesion to NETs-coated neutrophils, which was amplified by PMA and reversed by DNase I (Fig. 6E), indicating tumor-NETs crosstalk. Building on CREB1 K122la-HMGB1 interaction (Fig. 5I), HMGB1-neutralizing antibody abolished K122Q-conditioned medium-induced NETosis (Fig. 6F), definitively establishing HMGB1 as the essential mediator linking lactylation to NETs-driven immunosuppression.

### Exosomal HMGB1-TLR4 axis mediates NETosis-driven cisplatin resistance

**Figure 7:**
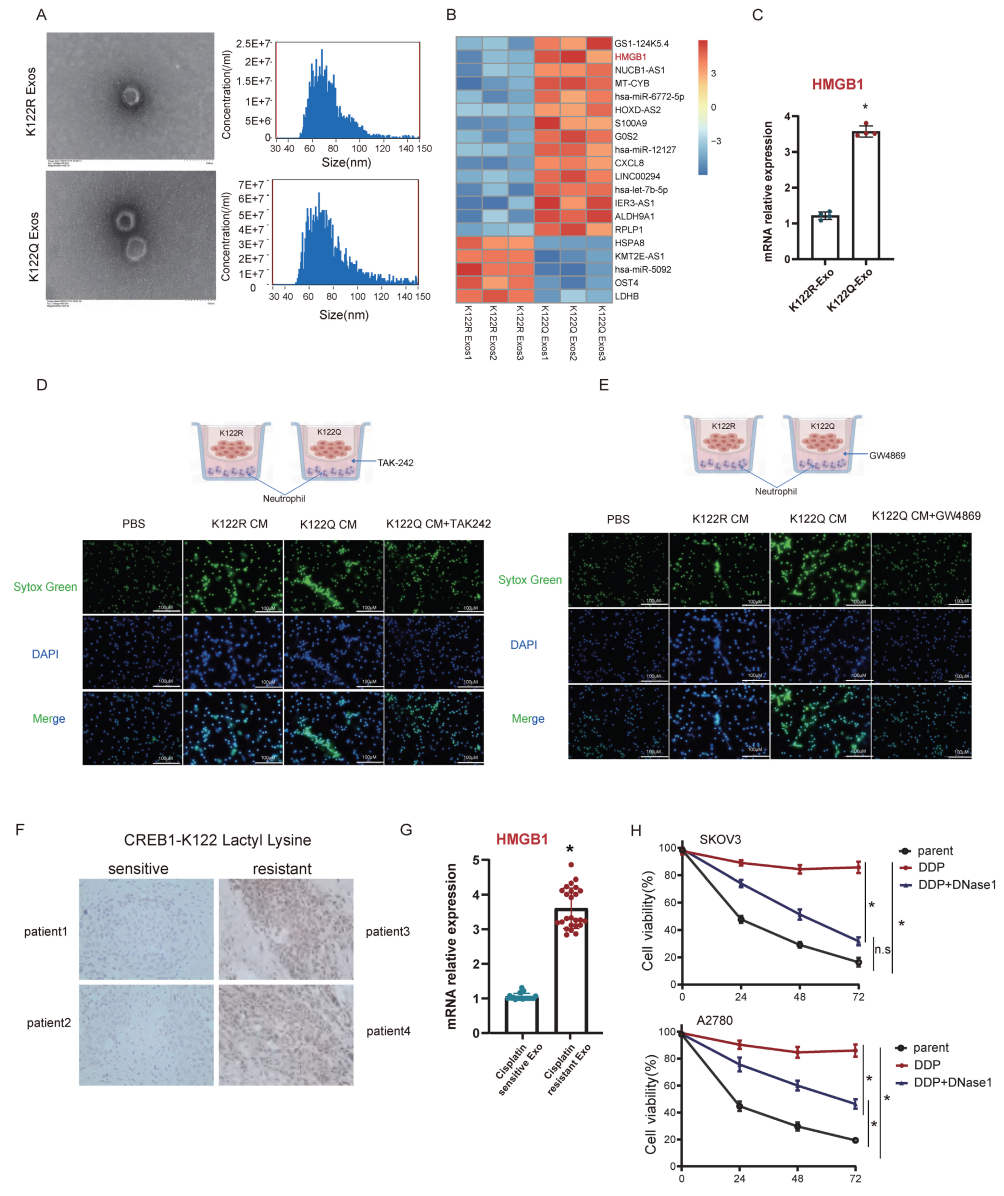
** Exosomal HMGB1-TLR4 axis mediates NETosis-driven cisplatin resistance.** A, Isolation and hydrodynamic diameter characterization of exosomes derived from K122R/K122Q Mutant Cells. B, Heatmap visualization of differentially expressed genes identified by transcriptomic profiling of Exosomes derived from K122R/K122Q Mutant Cells. C. RT-qPCR was used to detect the HMGB1 mRNA level in CREB1 K122R/K122Q cell's exosomes. Data presented as mean ± SD; * P < 0.05.D-E, Quantitative analysis of NETs formation in co-culture systems treated with GW4869 (Exosomal Inhibitor) and TAK-242 (TLR4 Signaling Inhibitor). F, Immunohistochemical analysis of CREB1-K122 Lactylation levels in pathological tissues from cisplatin-sensitive and cisplatin-resistant ovarian cancer patients. G, RT-qPCR analysis of HMGB1 expression in blood-derived exosomes from cisplatin-sensitive and cisplatin-resistant ovarian cancer patients (n = 25 per group). Data presented as mean ± SD; * P < 0.05.H, Chemoresistance profiling of ovarian cancer cell Lines following DNase1 (NETs inhibitor) treatment. Data presented as mean ± SD; * P < 0.05; n.s., not significant.

To define HMGB1's secretory mechanism, exosomes were isolated from K122Q and K122R mutant cells. Transcriptomics revealed selective enrichment of HMGB1 in K122Q-derived exosomes (Fig. 7A-B), confirmed by qPCR (Fig. 7C), indicating CREB1 lactylation as a direct regulator of exosomal HMGB1 sorting. To determine whether CREB1 K122la globally reprograms the exosomal cargo landscape or selectively enriches HMGB1, we examined a panel of exosomal cargo proteins by Western blot in exosomes isolated from K122R and K122Q cells. As shown in Supplementary Fig. 3, HMGB1 was consistently and markedly enriched in K122Q-derived exosomes, whereas the levels of other tested cargo proteins, including S100A8, IL-6, and the exosomal markers CD63, and TSG101 remained comparable between the two genotypes. These findings indicate that HMGB1 is a selectively enriched exosomal cargo, rather than a passive consequence of a globally altered exosomal secretion landscape.

While HMGB1-TLR4 signaling reportedly activates NETosis, its role in ovarian cancer chemoresistance was unknown. TLR4 inhibition using TAK-242 abrogated exosomal HMGB1-induced NETosis from K122Q mutants (Fig. 7D). In addition, blocking exosome secretion using GW4869 eliminated NETotic activity of K122Q-conditioned medium (Fig. 7E). The results indicate that HMGB1 is secreted extracellularly via the exosomal pathway. Clinically, cisplatin-resistant patients exhibited elevated tumor CREB1 lactylation (Fig. 7F) and serum exosomal HMGB1 levels (Fig. 7G, n = 25 per group), establishing this dual biomarker signature as clinically actionable for resistance monitoring. Treatment with the NETs inhibitor DNase1 significantly restored sensitivity of ovarian cancer cells to cisplatin (Fig. 7H), further implicating NET formation in chemoresistance of ovarian cancer. To further investigate the clinical relevance of the CREB1 K122la-HMGB1-NETs axis, we performed a correlation analysis between serum exosomal HMGB1 levels and serum NETs (MPO-DNA complexes) in the same cohort of 50 ovarian cancer patients (25 sensitive and 25 resistant). As shown in Supplementary Fig. 4, HMGB1 levels were positively correlated with NETs in the total cohort (r = 0.96, *P* < 0.001). Notably, this correlation was more pronounced in the cisplatin-resistant subgroup (r = 0.70, *P* < 0.001) compared with the sensitive subgroup (r = 0.41, *P* < 0.05). These data provide clinical evidence supporting that HMGB1 drives NETosis in patients, particularly in the context of chemoresistance.

### Targeted delivery of lactylation-deficient CREB1 K122R via lipid nanoparticles reverses cisplatin resistance *in vivo*

**Figure 8:**
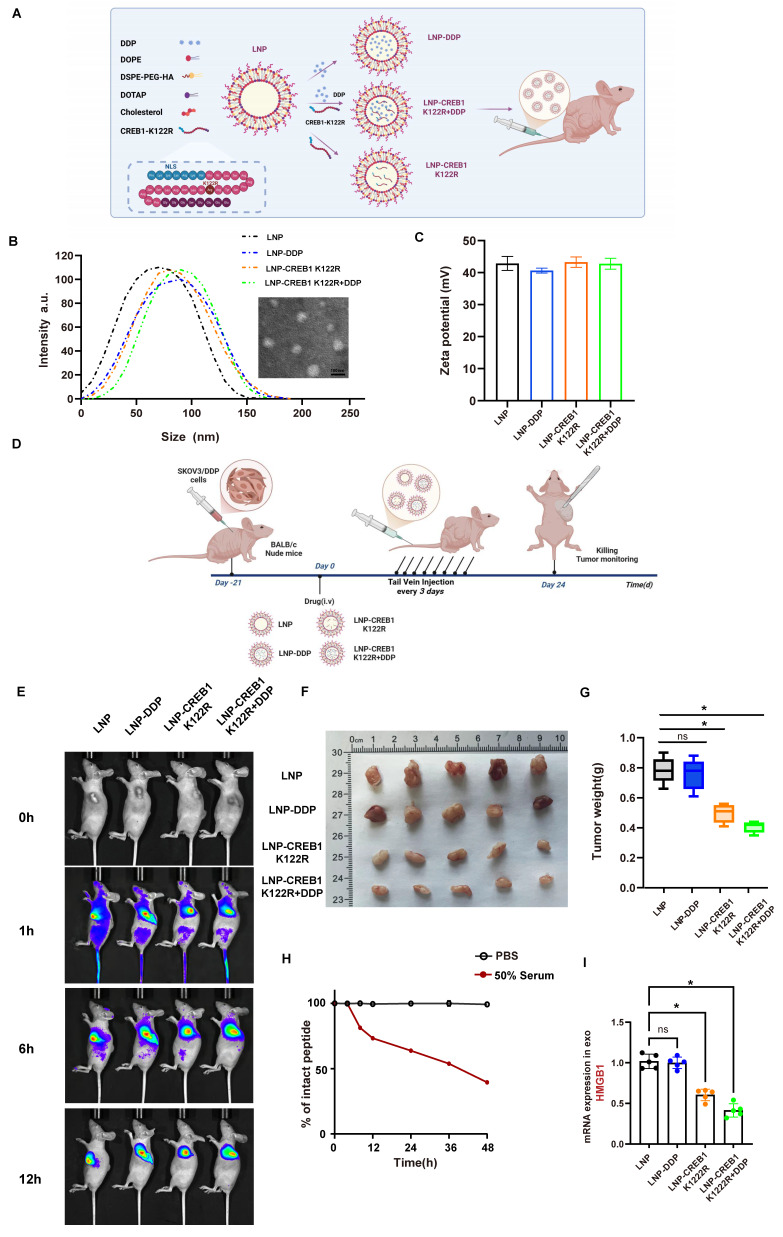
** Targeted delivery of lactylation-deficient CREB1 K122R via lipid nanoparticles reverses cisplatin resistance *in vivo*.** A, Schematic illustration of the tumor-targeting lipid nanoparticle (LNP) system co-loaded with the lactylation-deficient CREB1 K122R competitive peptide and cisplatin (DDP). The peptide is fused with a nuclear localization signal (NLS) for nuclear entry, and the LNP is decorated with an HA-tag for tumor targeting. B, Hydrodynamic diameter distribution of LNP formulations as measured by dynamic light scattering. C, Zeta potential values of the various LNP formulations. D, Experimental timeline for the *in vivo* therapeutic efficacy study in SKOV3/DDP xenograft models. E, *In vivo* fluorescence imaging showing systemic circulation and tumor-specific accumulation of Cy5.5-labeled LNPs at 1, 6, and 12 hours post-injection. F, Tumor weights from each treatment group.G, Tumor growth curves over the treatment period. H, The peptide was incubated at 37°C in 50% mouse serum (filled circles) or PBS alone (open circles) as a control. At the indicated time points, aliquots were removed and the remaining intact peptide was quantified by HPLC. I, Serum exosomal HMGB1 mRNA levels measured by qPCR after treatment.

To mechanistically counteract p300-mediated CREB1 activation, we rationally designed a lactylation-deficient CREB1 K122R competitive peptide (Fig. 8A). The critical K122 site was mutated to arginine (K-R), aiming to maintain binding specificity for p300 while rendering the peptide unmodifiable. As a result, this peptide acts as a competitive non-catalytic inhibitor, effectively occupying the active site of p300 in the nucleus. This action prevents p300 from accessing and lactylating endogenous full-length CREB1, thereby blocking the drug resistance pathway (Fig. 8A). Physicochemical characterization confirmed the successful preparation of the LNPs. Transmission electron microscopy revealed that the LNP-CREB1 K122R particles were spherical and monodisperse. Consistent with this, dynamic light scattering analysis showed a uniform hydrodynamic diameter of approximately 100 nm (Fig. 8B) and a favorable zeta potential (Fig. 8C).

We established SKOV3/DDP xenograft models and intravenously administered the following formulations every three days for 24 days: LNP, LNP-DDP, LNP-CREB1 K122R, and LNP-CREB1 K122R + DDP (containing both cisplatin and the CREB1 K122R competitive peptide). Subcutaneous tumors were harvested for analysis (Fig. 8D). *In vivo* imaging revealed that the LNP delivery system circulated systemically at 1 hour post-injection, accumulated at the tumor site by 6 hours, and showed complete tumor-specific localization by 12 hours, demonstrating high targeting efficiency (Fig. 8E). After 24 days, tumor analysis showed that LNP-DDP alone failed to suppress tumor growth, whereas LNP-CREB1 K122R monotherapy significantly inhibited tumor progression. The combination of LNP-CREB1 K122R with DDP resulted in the most substantial reduction in tumor weight (Fig. 8F-G). To evaluate the stability of the NLS-CREB1 K122R peptide under physiological conditions, we performed an *in vitro* serum stability assay. The peptide was incubated in 50% mouse serum at 37°C, and the remaining intact peptide was quantified by HPLC at indicated time points. The peptide exhibited gradual degradation in serum, while remaining stable in PBS control. Based on the degradation curve, the estimated half-life of the peptide was approximately 36 hours, supporting the every-three-day dosing regimen used in our *in vivo* studies (Fig. 8H). Furthermore, mice receiving the combination therapy exhibited a marked decrease in serum exosomal HMGB1 levels (Fig. 8I), indicating effective blockade of the HMGB1-mediated NETosis signaling axis.

We also evaluated the biosafety profile of the treatments by analyzing H&E-stained sections of key organs (heart, liver, spleen, lung, and kidney). The results demonstrated no apparent histopathological changes, such as inflammation, degeneration, or necrosis, across all groups. Furthermore, serum biochemical analysis revealed no significant differences in hepatic (ALT, AST) and renal (BUN, creatinine) function markers between the treatment and control groups, and flow cytometric analysis of peripheral blood showed no alteration in the T/B cell ratio, indicating preserved immune function (Supplementary Fig. 5), confirming the favorable *in vivo* safety of the LNP formulations at the therapeutic dose. To evaluate whether this competitive peptide interferes with CREB1 physiological function in non-cancerous cells, we examined its effect on canonical CREB1 target gene expression in human embryonic kidney 293 (HEK293) cells. Cells were treated with LNP-CREB1 K122R or empty LNP for 24 h, and the mRNA levels of c-Fos, Nr4a2, and Bcl-2 were quantified by qRT-PCR. As shown in Supplementary Fig. 5E, no significant differences in target gene expression were observed between the K122R peptide-treated group and the control groups, indicating that the peptide does not disrupt basal CREB1 transcriptional activity in normal cells. This specificity supports the therapeutic selectivity of our approach.

These results demonstrate that LNP-mediated delivery of the lactylation-deficient CREB1 K122R mutant effectively reverses CREB1 K122la-driven cisplatin resistance, providing a potential nanotherapeutic platform for clinical translation.

### Systems-level validation and pan-cancer prognostic value of the CREB1-HMGB1-NETosis axis

To ascertain whether the mechanistically defined lactate-CREB1-HMGB1-NETs axis operates as a coordinated transcriptional network in clinical settings, we performed Weighted Gene Co-expression Network Analysis (WGCNA) on ovarian cancer transcriptomic cohorts (Supplementary Fig. 6A). We identified a highly connected gene module strongly associated with therapy resistance. Network topology analysis revealed the core hub genes orchestrating this specific module (Supplementary Fig. 6B). Crucially, to distinguish direct epigenetic targets from secondary downstream effects, we overlaid our CUT&Tag-derived CREB1 binding targets with this resistance-associated WGCNA module. This intersection confirmed that lactylated CREB1 directly binds and regulates key hub effectors, most notably *HMGB1*, within this specific network (Supplementary Fig. 6C). Furthermore, Gene Ontology (GO) enrichment analysis of this hub-regulated network demonstrated a significant co-enrichment of glycolytic processes and neutrophil extracellular trap formation (Supplementary Fig. 6D). These systems-level findings validate that metabolic rewiring and immune evasion are not isolated events, but rather strictly coupled transcriptional programs driven directly by the CREB1/HMGB1 hubs.

To further evaluate the clinical predictive value of this axis, we performed survival analysis based on the expression of this newly identified metabolism-immunity signature. Patients exhibiting high expression of this hub-driven network experienced significantly worse clinical outcomes, including reduced overall survival (Supplementary Fig. 6E). This indicates that the CREB1-HMGB1-NETosis network serves as a robust prognostic indicator capable of stratifying patients at high risk of chemoresistance.

Finally, given that profound metabolic reprogramming is a hallmark of multiple treatment-refractory malignancies, we explored the broader clinical relevance of this axis beyond ovarian cancer. Focusing strictly on renal cell carcinoma (RCC), a tumor type distinguished by extensive metabolic plasticity and mitochondrial adaptation, we successfully reconstructed the WGCNA module in the RCC transcriptomic cohorts (Supplementary Fig. 6F) and validated the conserved core hub network architecture (Supplementary Fig. 6G). More importantly, to address the clinical relevance across different RCC metabolic landscapes, we evaluated the prognostic value of this conserved module. Strikingly, high expression of the CREB1-HMGB1-NETosis module was consistently and significantly associated with poorer overall survival across all three major RCC subtypes: Kidney renal clear cell carcinoma (KIRC, Supplementary Fig. 6H), Kidney Chromophobe (KICH, Supplementary Fig. 6I), and Kidney renal papillary cell carcinoma (KIRP, Supplementary Fig. 6J).

To further dissect the intersection between this conserved resistance module and specific metabolic features in RCC, we evaluated its correlation with lactate metabolism, mTOR signaling, and mitochondrial dynamics. We found that the module signature expression exhibited a significant positive correlation with the expression of key lactate metabolism genes, including *LDHA* (r = 0.323, *P* < 0.001), *MCT1* (*SLC16A1*; r = 0.300, *P* < 0.001), and *MCT4* (*SLC16A3*; r = 0.396, *P* < 0.001) (Supplementary Fig. 6K). Furthermore, Gene Set Variation Analysis (GSVA) revealed a highly significant positive correlation between the module signature and the mTOR signaling score (r = 0.532, *P* < 0.001), indicating that this module is tightly linked to mTOR activation (Supplementary Fig. 6L). Concurrently, consistent with a metabolic shift toward glycolysis, high module expression was significantly associated with reduced mitochondrial gene expression (r = -0.696, *P* < 0.001) and lower mtDNA copy number estimated from sequencing data (r = -0.791, *P* < 0.001) (Supplementary Fig. 6M-N). Together, these analyses demonstrate that the CREB1-HMGB1-NETosis network is deeply embedded within a broader metabolic reprogramming landscape, interacting synergistically with mTOR-driven glycolysis and mitochondrial dysfunction.

These findings suggest that the lactate-CREB1-HMGB1-NETosis axis represents a highly conserved resistance-associated transcriptional program, establishing its potential as a robust predictive biomarker and therapeutic target in metabolically reprogrammed tumors.

## Discussion

This study established CREB1 K122la as a previously unrecognized epigenetic driver of cisplatin resistance in ovarian cancer, dynamically regulated by the opposing enzymatic activities of p300 (writer) and SIRT1 (eraser). Critically, CREB1 K122la orchestrates extensive transcriptional reprogramming that activates exosomal secretion of HMGB1. This secreted alarmin engages TLR4 on tumor-associated neutrophils to initiate NET formation, thereby establishing an integrated lactate-CREB1 K122la-HMGB1-NETs axis. Our findings decode a novel tripartite metabolic-epigenetic-immune crosstalk underlying chemoresistance^37^, ^38^, fundamentally expanding understanding of resistance mechanisms beyond canonical DNA repair pathways.

Unlike established CREB1 regulation via phosphorylation or acetylation^21^, ^39^, we pioneer the discovery of lactylation-dependent epigenetic rewiring. This modification dramatically remodels chromatin architecture, expanding chromatin accessibility and recruiting greater repertoire of target genes compared to lactylation-deficient controls. Mechanistically, K122la redistributes CREB1 binding from proximal promoters toward distal enhancer regions, a distinct regulatory paradigm contrasting with histone lactylation-mediated metabolic activation in macrophages 10 or MRE11 lactylation-mediated DNA repair in breast cancer^9^.

While lactylation broadly influences oncogenic phenotypes^40^, ^41^, our work uniquely identifies a non-histone lactylation event that actively redirects transcriptional networks toward NETosis-mediated immune evasion. Although neutrophil function exhibits context-dependent roles in therapy response 27, 42, 43, our data definitively implicate CREB1 K122la-driven NETosis as a conserved resistance mechanism across malignancies. Notably, HMGB1, while previously recognized as a NETosis inducer 44, 45, is newly identified as a chemoresistance effector transcriptionally controlled by lactylated CREB1. While our data establish that CREB1 K122la transcriptionally upregulates HMGB1 and that exosomal HMGB1 is required for NETosis, the precise mechanisms linking HMGB1 transcription to its preferential exosomal loading remain to be fully elucidated. Potential contributing factors, such as HMGB1 post-translational modifications or interaction with exosomal sorting machineries, warrant future investigation.

In the present study, we demonstrate that CREB1 K122la directly binds the HMGB1 promoter and enhances its transcription (Fig. 5J-K; Fig. 5L-M), and that HMGB1 protein is selectively enriched in exosomes from K122Q-expressing cells (Fig. 7A-C). To dissect the functional contribution of extracellular versus intracellular HMGB1, we employed an HMGB1-neutralizing antibody that specifically blocks extracellular alarmin function. This antibody phenocopied the effects of exosome inhibition (GW4869) and TLR4 blockade (TAK242) in abrogating K122Q-driven NETosis and chemoresistance (Fig. 6F, 7D-E, 7H), supporting the conclusion that extracellular, exosomal HMGB1 acts as a critical downstream effector. Although HMGB1 also exerts well-established intracellular functions such as transcriptional co-activation and autophagy regulation, our data collectively suggest that in the context of CREB1 K122la-driven cisplatin resistance, the alarmin function of HMGB1, rather than its intracellular transcriptional activity, constitutes the predominant mechanism underlying NETosis.

The identification of p300 and SIRT1 as the writer and eraser of CREB1 K122 lactylation raises the possibility that their pleiotropic effects might contribute to broader chromatin changes beyond the modulation of this specific site. However, our genetic rescue experiments in CREB1-knockout cells reconstituted with lactylation-deficient (K122R) or lactylation-mimetic (K122Q) mutants largely circumvent this concern. In this isogenic system, endogenous p300 and SIRT1 activities remain unchanged, yet the K122R and K122Q mutants confer markedly different chromatin binding (CUT&Tag), transcriptional outputs, and cisplatin responses (Fig. 5). This demonstrates that the modification state of K122 is sufficient to drive the observed phenotypes independently of global changes in p300 or SIRT1 activity. Nevertheless, we cannot exclude potential contributions from lactylation of other substrates, a possibility that warrants future investigation, such as chromatin-wide lactylation profiling following selective modulation of CREB1 K122 lactylation.

We acknowledge that our CUT&Tag data alone do not definitively distinguish whether CREB1 K122la actively remodels chromatin or preferentially binds pre-existing open enhancers. Direct approaches such as ATAC-seq or biophysical binding assays will be required to fully resolve this mechanism, and we are actively pursuing these studies.

We also acknowledge that the proposed competitive mechanism by which the K122R peptide prevents p300 from accessing and lactylating endogenous CREB1 is inferred from in vivo data rather than direct biochemical evidence. While our findings demonstrate that K122 is functionally critical for CREB1 lactylation and that the K122R peptide reverses chemoresistance, these observations do not formally prove steric hindrance of p300 binding or enzymatic activity. Future studies using purified components and in vitro lactylation assays will be required to definitively establish the molecular mechanism.

Furthermore, our study extends beyond mechanistic revelation to therapeutic exploration. The successful development of a tumor-targeting lipid nanoparticle (LNP) system for the delivery of a lactylation-deficient CREB1 K122R competitive peptide (Fig. 8A-C) represents a significant translational advance. The high tumor-specific accumulation of our LNP construct (Fig. 8E) validates its targeting efficacy and provides a promising platform for precise intervention. Most importantly, the combination of LNP-CREB1 K122R with cisplatin potently suppressed tumor growth *in vivo* (Fig. 8F-H), demonstrating that direct targeting of the CREB1 K122la axis can effectively reverse established cisplatin resistance. The concomitant decrease in serum exosomal HMGB1 levels in the combination treatment group (Fig. 8I) provides compelling evidence that the therapeutic efficacy operates through the specific disruption of the lactate-CREB1 K122la-HMGB1-NETosis cascade we have delineated.

Therapeutically, our mechanistic findings nominate actionable nodes within the lactate-CREB1 K122la-HMGB1-NETs axis for clinical intervention. Pharmacological inhibition of p300, the identified writer enzyme of K122la, using clinical-grade inhibitors such as A485^46^, ^47^ represents a promising strategy, particularly given demonstrated preclinical efficacy of A485 in abrogating p300-medaited oncogenic transcription in multiple tumors^48^-^50^, which supports its repurposing potential for cisplatin-resistant ovarian cancer. Complementary approaches include neutralizing secreted HMGB1 51 or blocking its extracellular signaling through TLR4 antagonists like TAK-242^52^, the latter having advanced to Phase II trials for inflammatory diseases^53^, ^54^, thereby accelerating its translational applicability. Further downstream, direct disruption of NETs structures via DNase I or novel peptidyl arginine deiminase 4 (PAD4) inhibitors 55, 56 may synergize with platinum-based chemotherapy by dismantling the chemoprotective niche. Importantly, rational combinatorial approaches, such as concurrent p300 inhibition and NETs destabilization, hold significant potential to circumvent compensatory resistance pathways and achieve durable therapeutic response.

This nanotherapeutic approach offers several advantages. First, by using a competitive short peptide, it directly interferes with the protein-protein interaction or lactylation modification interface, potentially yielding higher specificity than broad-spectrum enzymatic inhibitors. Second, the NLS-fused peptide design ensures nuclear localization, enabling direct engagement with chromatin-bound CREB1 and its transcriptional machinery. Third, the LNP-mediated co-delivery of the K122R peptide and cisplatin embodies a rational combination strategy that simultaneously targets the epigenetic driver and the conventional chemotherapeutic agent, thereby attacking the resistance mechanism from multiple angles.

Clinically, we propose a dual biomarker signature with elevated tumor K122 lactylation and serum exosomal HMGB1, that effectively stratifies cisplatin resistance. While our findings with the LNP-CREB1 K122R platform are promising, several considerations remain for future development. The long-term stability, potential immunogenicity, and comprehensive biosafety profile of this nanofomulation warrant further investigation in advanced models. It will also be crucial to explore whether this strategy synergizes with other emerging modalities, such as immune checkpoint inhibitors, given the involvement of NETosis in shaping the immunosuppressive TME. Formal long-term toxicity and pharmacokinetic studies are underway to further support the clinical translation of this LNP-based strategy. Nonetheless, our work establishes the proof-of-concept that targeting CREB1 lactylation via a nanocarrier-delivered decoy peptide is a viable and potent strategy to overcome chemoresistance. While our immunodeficient models preclude assessment of humanized immune interactions and biomarker validation requires larger cohorts (n>30), this non-invasive approach holds significant diagnostic potential.

Priority research directives emerging from this work encompass three critical translational pathways: (1) Developing clinical-grade anti-K122la antibodies represents an essential foundation for precise detection and stratification of lactylation-driven resistance. (2) Systematic evaluation of p300/HMGB1/NETs inhibitor combinations in patient-derived xenograft (PDX) models is warranted to delineate synergistic therapeutic efficacy. (3) Advancing first-in-class NETosis blockers, including optimized DNase formulations and novel PAD4 inhibitors, constitutes a pivotal frontier for dismantling chemoprotective niches.

Regarding the metabolic regulation of this axis, our pharmacological data firmly establish that glycolytic flux is indispensable for CREB1 lactylation. Intracellular lactate canonically acts as a direct metabolic substrate via rapid conversion to lactyl-CoA. However, we cannot rule out the possibility that extracellular lactate might also function concurrently as a signaling molecule (e.g., via receptors like GPR81) to synergistically amplify the lactylation machinery. A limitation of our current study is the reliance on robust enzymatic inhibition rather than direct stable isotope tracing. Future 13C metabolic flux analyses via specialized LC-MS/MS are required to definitively map the exact biochemical pathways driving the accumulation of this lactyl group.

Furthermore, our cross-cancer extended analysis reveals that the CREB1-mediated “Glycolysis-NETosis” network is highly conserved and carries significant prognostic value across major subtypes of renal cell carcinoma (RCC), a tumor classically characterized by profound metabolic plasticity. Recent innovative network-based studies have highlighted the paramount importance of integrating co-expression modules with immune and metabolic landscapes to identify robust pan-cancer biomarkers^35^. Inspired by this conceptual framework, while our current multi-omics study successfully establishes the prognostic utility of this network in RCC, its deeper mechanistic intersection with specific metabolic features, such as mTOR signaling activation and mitochondrial dynamics remains a fascinating and critical area for future prospective exploration. Finally, we acknowledge that the predictive biomarker value in our current study is primarily derived from retrospective cohorts. Future prospective, longitudinal clinical trials will be essential to fully translate this systems-level network signature into therapeutic practice.

Collectively, targeting the lactate-CREB1 K122la-HMGB1-NETs axis provides a potential precision oncology framework to overcome cisplatin resistance through coordinated metabolic modulation, epigenetic reprogramming, and immune microenvironment remodeling.

## Conclusions

Our study identifies the lactate-CREB1 K122la-HMGB1-NETs axis as a key driver of cisplatin resistance in ovarian cancer, integrating metabolic, epigenetic, and immune mechanisms. CREB1 lactylation at K122-regulated by the opposing activities of p300 and SIRT1-reprograms transcriptional networks, enhancing HMGB1 expression and promoting its exosomal secretion. Secreted HMGB1 triggers TLR4-dependent NETosis, establishing a chemoprotective niche that shields tumor cells from cisplatin.

Critically, we demonstrate the therapeutic potential of targeting this axis through the development of a tumor-targeted lipid nanoparticle system delivering a lactylation-deficient CREB1 K122R competitive peptide. This nanotherapeutic strategy effectively reversed cisplatin resistance *in vivo*, with combination therapy achieving significant tumor suppression accompanied by reduced serum exosomal HMGB1 levels.

These findings not only decipher a novel metabolic-epigenetic-immune circuitry underlying chemoresistance but also provide a promising nanomedicine-based strategy to restore chemosensitivity. The dual biomarker-tissue CREB1 K122la and serum exosomal HMGB1, offer clinical potential for resistance stratification, while the LNP-mediated targeting approach presents a viable path toward overcoming chemoresistance in ovarian cancer patients.

## Supplementary Material

Supplementary figures.

## Data Availability

All data in this study will be available from the corresponding author on reasonable request.

## Abbreviations

DEGs: differentially expressed genes
ECAR: extracellular acidification rate
HMGB1: high mobility group box 1
IC₅₀: half-maximal inhibitory concentration
Kla: lysine lactylation
LNP: lipid nanoparticle
NETs: Neutrophil extracellular traps
NLS: nuclear localization signal
OCR: oxygen consumption rate
PAD4: peptidyl arginine deiminase 4
PDX: patient-derived xenograft
PTM: post-translational modification
TLR4: Toll-like receptor 4
TME: tumor microenvironment
