# Supplementary material for: Reprogramming Transcriptional Networks via CREB1 Lactylation at K122 Activates HMGB1-Mediated NETosis and Chemoresistance
Source: Int J Biol Sci. 2026 May 18;22(10):5568–94. doi: 10.7150/ijbs.131303 (PMC13215458; doi:10.7150/ijbs.131303)

Supplementary Figure 1：Glycolytic inhibition by 2-DG reduces glucose consumption, lactate production, and CREB1 lactylation in cisplatin-resistant ovarian cancer cells A-B, Glucose consumption (A) and lactate production (B) in SKOV3/DDP and A2780/DDP cells treated with vehicle or 2-DG (5 mM, 24 h). Data are presented as mean ± SD. * $P < 0.05$. C, Co-immunoprecipitation (Co-IP) analysis of CREB1 lactylation in SKOV3/DDP and A2780/DDP cells treated with vehicle or 2-DG. Cell lysates were immunoprecipitated with anti-pan-Kla antibody, followed by immunoblotting with anti-CREB1 to detect lactylated CREB1. D, SKOV3 cells were treated with sodium lactate (20 mM) alone or in combination with acetate (20 mM) or butyrate (20 mM) for 24 h. LDHi (10 mM, 48 h) was used as a control. Co-IP was performed with anti-pan-Kla antibody, followed by immunoblotting with anti-CREB1.E, SKOV3 cells were treated with sodium lactate (20 mM) or sodium pyruvate (20 mM) for 24 h. Co-IP analysis of CREB1 lactylation was performed as described in (D).

Supplementary Fig. 2. Motif enrichment analysis confirms sequence-specific binding of K122Q at enhancer regions. Motif analysis was performed using HOMER (v4.11). Target regions are K122Q-bound enhancers; background is a size-matched set of random genomic regions. The CRE motif is the most significantly enriched motif .

Supplementary Figure 3. Selective enrichment of HMGB1 in exosomes from K122Q-expressing cells. Western blot analysis of exosomal cargo proteins in exosomes isolated from K122R and K122Q cells.

Supplementary Fig. 4. Correlation between serum exosomal HMGB1 and serum NETs in ovarian cancer patients. Scatter plots showing the correlation between serum exosomal HMGB1 levels (measured by qRT-PCR) and serum NETs levels (MPO-DNA complexes, measured by ELISA) in 50 ovarian cancer patients. (A) Total cohort (n = 50) showing a significant positive correlation ($r = 0.96$, $P < 0.05$, Pearson correlation). (B) Cisplatin-sensitive subgroup (n = 25) showing a positive correlation ($r = 0.41$, $P < 0.05$). (C) Cisplatin-resistant subgroup (n = 25) showing a strong positive correlation ($r = 0.70$, $P < 0.05$).

Supplementary Figure. 5 .*In vivo* biosafety evaluation of LNP formulations. A, Representative H&E-stained sections of major organs (heart, liver, spleen, lung, kidney) showing no apparent histopathological changes across all treatment groups. B-C, Serum levels of hepatic (ALT, AST) and renal (BUN, creatinine) function markers measured at the end of treatment. D, Ratio of $CD3^+$ T cells to $CD19^+$ B cells in peripheral blood assessed by flow cytometry. Data are presented as mean ± SD (n = 5 per group) . E, Specificity of the K122R peptide in HEK293 cells. HEK293 cells were treated with empty LNP or LNP-CREB1 K122R for 24 h, and mRNA levels of canonical CREB1 target genes (c-Fos, Nr4a2, Bcl-2) were quantified by qRT-PCR. Data are presented as mean ± SD (n = 3). No significant differences were observed, indicating that the peptide does not interfere with basal CREB1 transcriptional activity.

Supplementary Figure 6. Systems-level integration of the lactylated CREB1-driven co-expression network and its pan-RCC prognostic value.A, Weighted Gene Co-expression Network Analysis (WGCNA) identifying the therapy resistance-associated gene module in the ovarian cancer transcriptomic cohort.B, Network topology visualization highlighting the core hub genes (including *CREB1* and *HMGB1*) within the identified resistance module. C,Venn diagram illustrating the highly specific intersection between lactylated CREB1 binding target genes (identified via CUT&Tag) and the WGCNA module genes, confirming the direct transcriptional regulation of key hub effectors. D, Gene Ontology (GO) functional enrichment analysis of the hub-

regulated network, demonstrating significant co-enrichment in glycolytic processes and neutrophil extracellular trap (NET) formation. E, Kaplan-Meier overall survival curve of ovarian cancer patients stratified by the expression signature score of the core hub network. F, Reconstruction of the resistance-associated WGCNA module in the Renal Cell Carcinoma (RCC) cohort from TCGA. G, Network visualization showing the highly conserved core hub architecture operating in RCC.H-J,Kaplan-Meier overall survival analyses evaluating the robust prognostic value of the conserved CREB1-HMGB1-NETosis module signature across three major RCC subtypes: (H) Kidney renal clear cell carcinoma (KIRC), (I) Kidney Chromophobe (KICH), and (J) Kidney renal papillary cell carcinoma (KIRP). Statistical significance was determined using the log-rank test. K, Spearman correlation analysis between the module signature and the expression of key lactate metabolism genes (LDHA, MCT1/SLC16A1, MCT4/SLC16A3) in RCC. L, Scatter plot demonstrating a significant positive correlation between the module signature and the mTOR signaling GSVA score. M-N, Scatter plots revealing a strong negative correlation between the module signature and both mitochondrial feature GSVA score (M) and mtDNA copy number (N)

Supplementary Figure 1

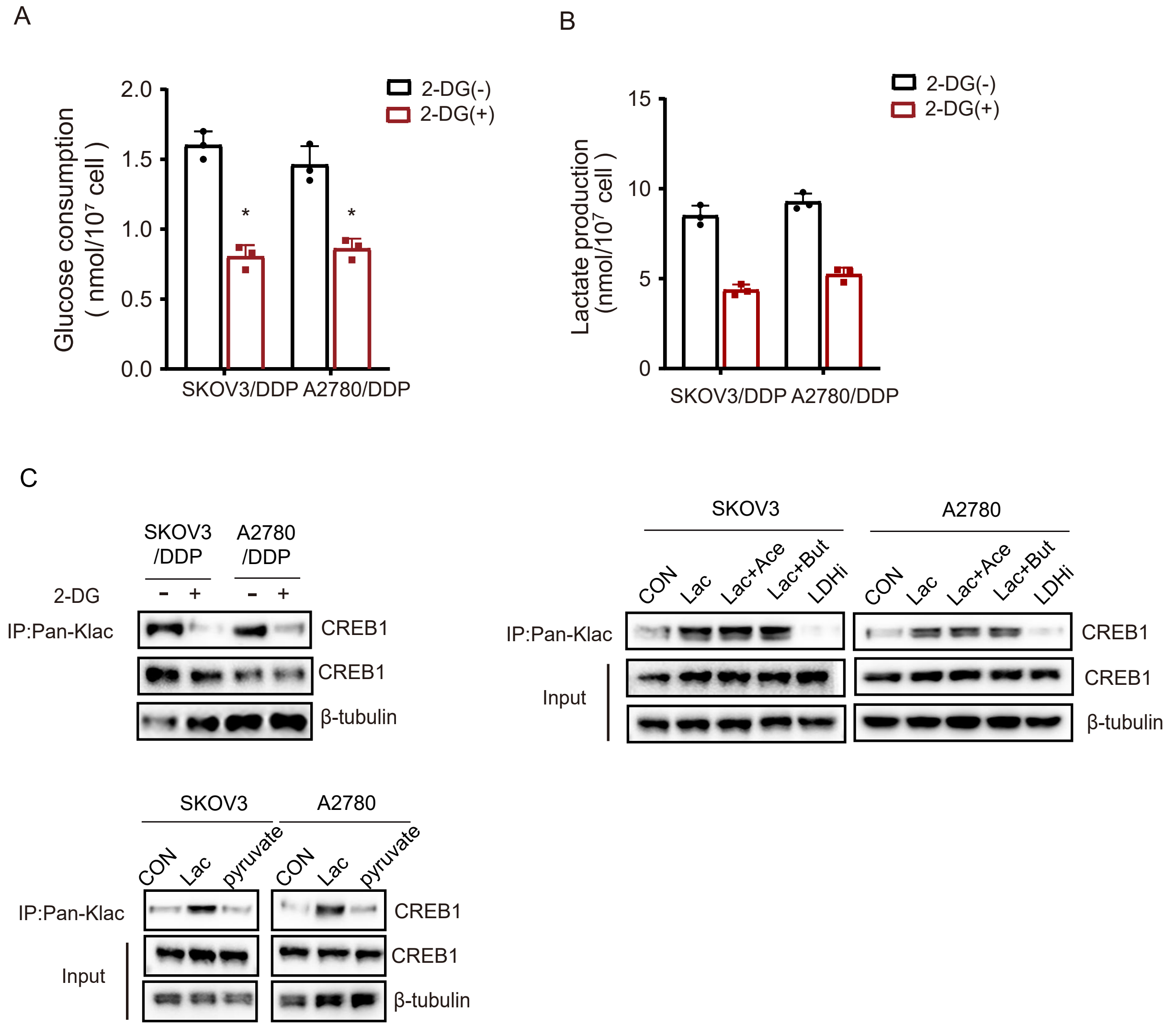

Supplementary Figure 2

Motif enrichment analysis of K122Q-bound enhancers

Rank	Motif	Best match	P value	% of targets	% of background	Fold Enrichment
1	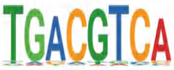 CRE	CREB1	1.0×10^{-125}	42.5%	3.1%	13.7
2	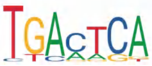 AP1	JUN	2.3×10^{-8}	18.3%	8.1%	2.3
3	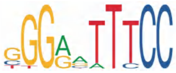 NF-κB	RELA	1.1×10^{-4}	8.7%	4.2%	2.1

Supplementary Figure 3

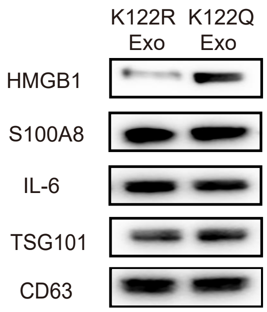

Supplementary Figure 4

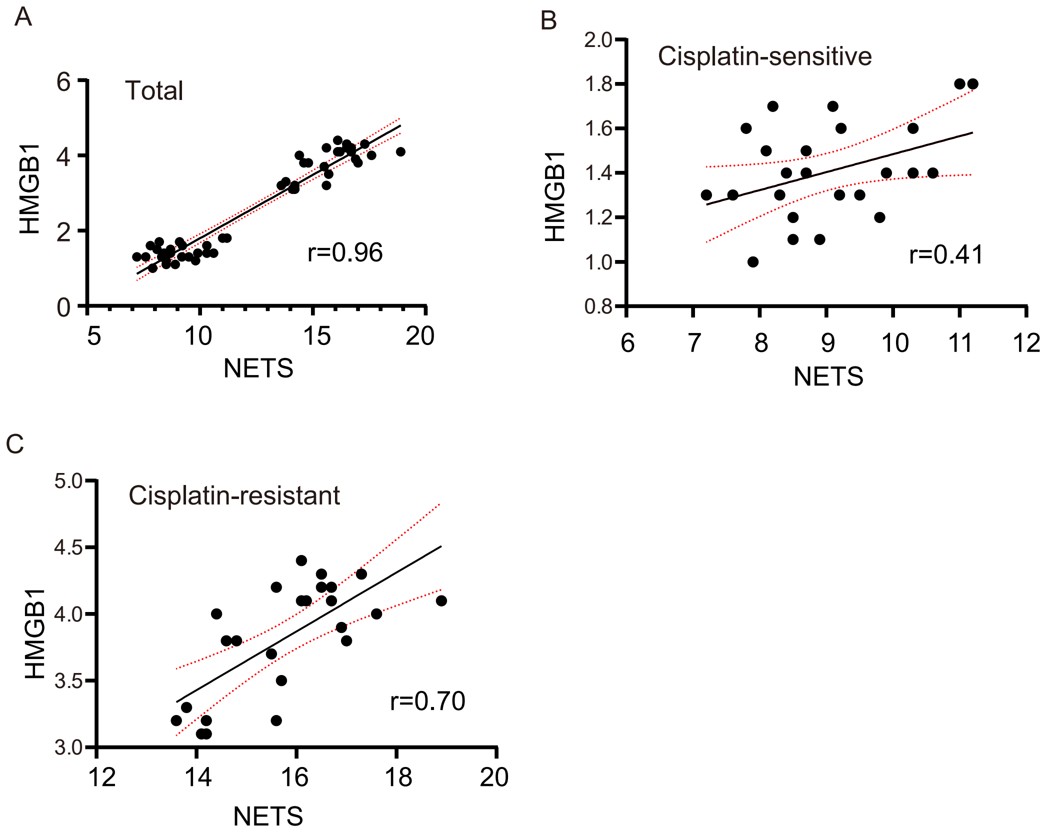

Supplementary Figure 5

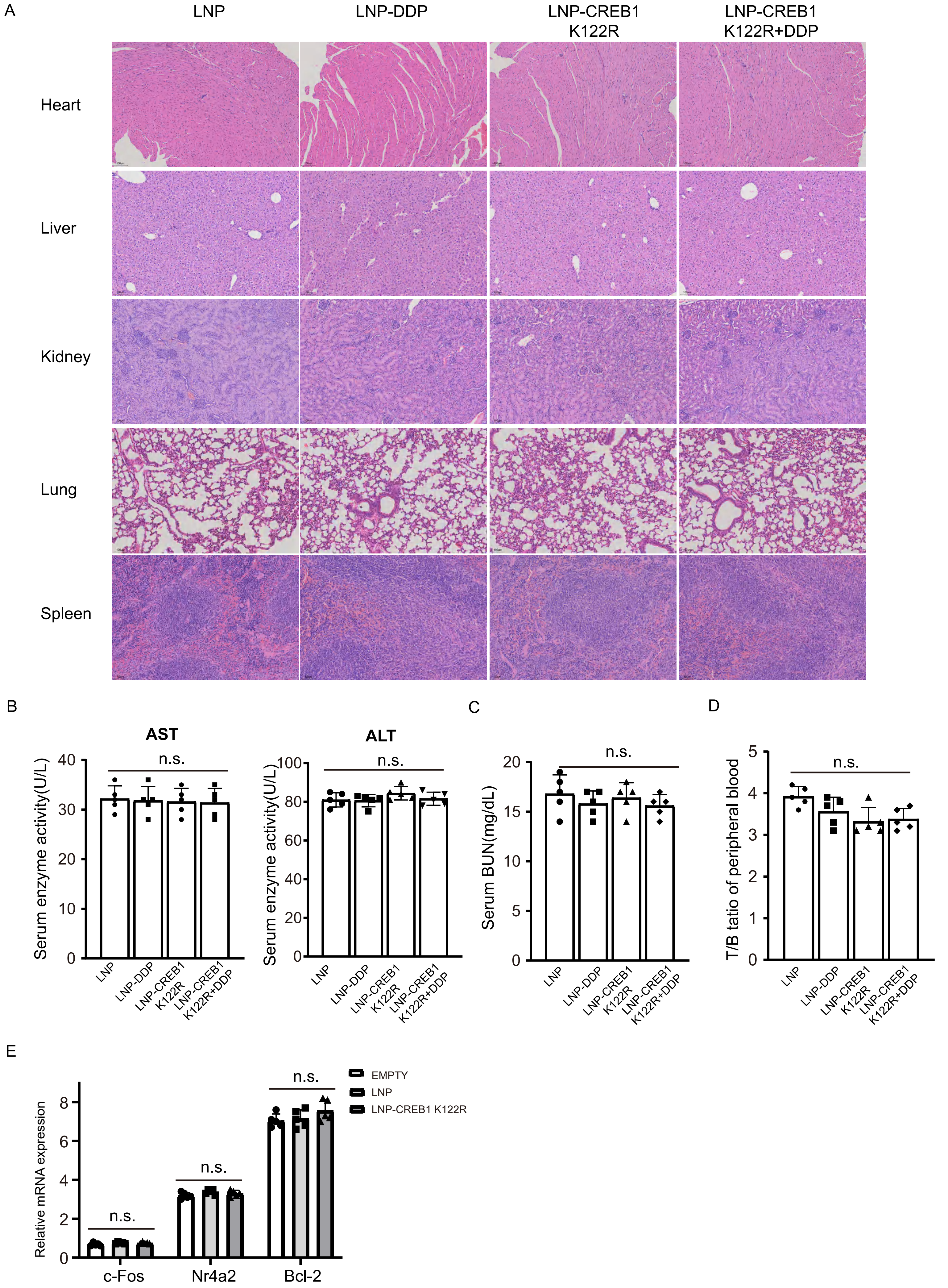

Supplementary Figure 6

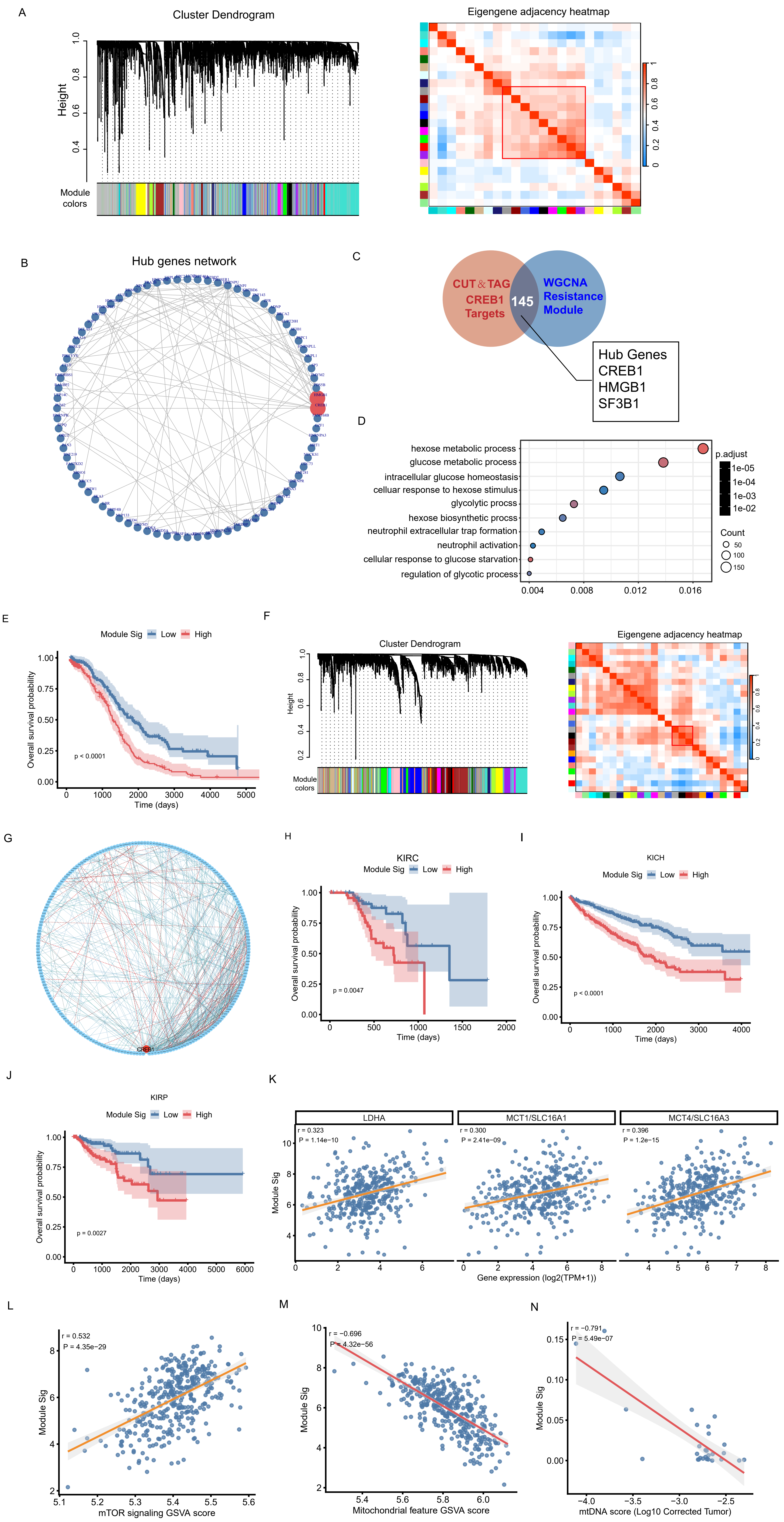

Supplement: Supplementary file 1 — Supplementary figures. [file ijbsv22p5568s1.pdf]
